# An innovative fractional-order evolutionary game theoretical study of personal protection, quarantine, and isolation policies for combating epidemic diseases

**DOI:** 10.1038/s41598-024-61211-2

**Published:** 2024-06-24

**Authors:** Masuda Akter, Nurunnahar, Mohammad Sharif Ullah, Mutum Zico Meetei, Abdullah A. Zaagan, Ali M. Mahnashi

**Affiliations:** 1 Department of Mathematics, Feni University, Feni, 3900 Bangladesh; 2 Department of Mathematics, College of Science, Jazan University, 45142 Jazan, P.O. Box 114, Kingdom of Saudi Arabia

**Keywords:** Evolutionary game, Fractional-order, Protection, Non-pharmaceutical intervention, Immunology, Diseases, Mathematics and computing

## Abstract

This study uses imposed control techniques and vaccination game theory to study disease dynamics with transitory or diminishing immunity. Our model uses the ABC fractional-order derivative mechanism to show the effect of non-pharmaceutical interventions such as personal protection or awareness, quarantine, and isolation to simulate the essential control strategies against an infectious disease spread in an infinite and uniformly distributed population. A comprehensive evolutionary game theory study quantified the significant influence of people’s vaccination choices, with government forces participating in vaccination programs to improve obligatory control measures to reduce epidemic spread. This model uses the intervention options described above as a control strategy to reduce disease prevalence in human societies. Again, our simulated results show that a combined control strategy works exquisitely when the disease spreads even faster. A sluggish dissemination rate slows an epidemic outbreak, but modest control techniques can reestablish a disease-free equilibrium. Preventive vaccination regulates the border between the three phases, while personal protection, quarantine, and isolation methods reduce disease transmission in existing places. Thus, successfully combining these three intervention measures reduces epidemic or pandemic size, as represented by line graphs and 3D surface diagrams. For the first time, we use a fractional-order derivate to display the phase-portrayed trajectory graph to show the model’s dynamics if immunity wanes at a specific pace, considering various vaccination cost and effectiveness settings.

## Introduction

Amidst the ongoing fight against epidemic diseases that substantially threaten worldwide public health, formulating efficient approaches for personal protection or awareness, quarantine, and isolation policy with vaccination programs is a crucial and challenging task. It is necessary to use creative and adaptable strategies due to their intricate and ever-changing nature to minimize the effect of infectious diseases on society. This study aims to enhance the ongoing discussion by providing a thorough comparative analysis of non-pharmaceutical measures, namely, personal awareness, quarantine, and isolation policies. It utilizes a unique ABC fractional-order modeling approach combined with evolutionary game theory, where government forces to participate in vaccination programs are also considered.

As shown by previous pandemics^1^, the pressing nature of the current global health crises emphasizes the need for a sophisticated comprehension of the interaction between personal preventive measures and more comprehensive social actions. The suggested technique for modeling incorporates fractional calculus, a mathematical tool that captures non-integer order dynamics^2–5^. This addition enhances the analysis by offering a more realistic depiction of the complex dynamics involved in the transmission and control of epidemics^6–8^. Dhar et al.^9^ provide valuable insights into the effectiveness of the ABC non-singular kernel fractional methods for both lenient and critical scenarios. Yadav et al.^6^ provide and examine a fractional order model of diabetes mellitus. They use the ABC derivative to quantitatively characterize and evaluate diabetes mellitus while excluding the effects of genetic factors. Yadav et al.’s^10^ analysis of a fractional model of the Ebola virus suggests that this novel technique could provide previously undiscovered insights into the Ebola viral model. Preventive interventions considerably restrict the transmission of measles in the population, according to research by Peter al.^11^, who present a unique mathematical framework to examine the dynamics of disease propagation. Farman et al.^12^ developed and assessed a fractional-order COVID-19 epidemic model, including quarantine and social distancing measures. They assert that their approach is practical for governmental control of disease transmission in a realistic manner.

In addition, the research incorporates evolutionary game theory^13–15^, a robust conceptual framework developed from game theory^16^, to analyze the strategic interactions between people and the changing dynamics of protective behaviors, personal awareness, adherence to quarantine measures, and compliance with isolation procedures. This method tries to gain insights into the processes determining the success of different intervention measures, considering the dynamic character of human behavioral reactions to changing epidemic settings. Zhou et al.^17^ demonstrated that implementing stringent government policies may effectively reduce the likelihood of infection, as determined using game-theoretic epidemiological analysis. Augsburger et al.^18^ scrutinize the impact of an imperfect vaccine on the results of a vaccination game using a basic SIR compartmental model to represent the spread of the disease. Kabir^19^ systematically examines several models of vaccination efficacy and vaccine contact reduction. From an evolutionary standpoint, Kabir^20^ investigates a dynamic vaccination game model incorporating a dyadic game and vaccine cost-effectiveness in an epidemic where cooperation among individuals appears to exist. A ‘free rider’ creates a feedback loop between disease prevalence and strategic individual vaccination behavior, according to an evolutionary game theory-based model analyzed by Bauch^21^. Khan^22^ proposed an epidemiological model that integrates SEIR dynamics and two kinds of interventions, voluntary self-isolation and mandatory quarantine while considering the dynamics of human behavior. Aronna et al.^23^ demonstrate that isolation (social distance) and testing asymptomatic patients are essential to controlling the pandemic, and the tighter these measures, the flatter the infection curve. Meijere^24^ states that isolating infected people is a crucial public health tool for managing the spread of communicable infections. Kabir et al.^25^ assert that wearing a mask may decrease disease transmission by reducing the spread of the virus from infected persons to others who are susceptible. Amaral et al.^26^ proposed an epidemiological SIR model incorporating evolutionary game theory to study disease dynamics. This model integrates social tactics, individual risk perception, and viral propagation into a unified process.

According to the abovementioned research, no work considering personal awareness, quarantine, and isolation policy has been done in the same context through the fractional-order derivative model with an EGT setting. To fill the gap, we proposed the current comparative study for the first time to examine the advantages and drawbacks of said strategies with vaccination games incorporating government forces to participate in vaccination programs, providing insight into their distinct effects on epidemic control. This analysis offers valuable perspectives on the efficacy of several intervention strategies, including personal protective measures, quarantine protocols, and isolation rules, which play a pivotal role in mitigating the transmission of infectious diseases. In the fractional-order viewpoint and evolutionary game theory context, this multidisciplinary research enhances the theoretical comprehension of epidemic control. It enables a more nuanced analysis of the evolutionary dynamics, which has the potential to provide more customized and efficient policy suggestions for the control and alleviation of diseases due to the memory effects. It offers practical perspectives to guide policymakers, public health authorities, and researchers in devising more resilient and flexible ways to fight against infectious diseases. Therefore, we are banking on our numerical results, which will afford valuable insights for policymakers and public health authorities in formulating proactive and adaptable approaches to address epidemics and protect the population.

## Model and methods

**Figure 1:**
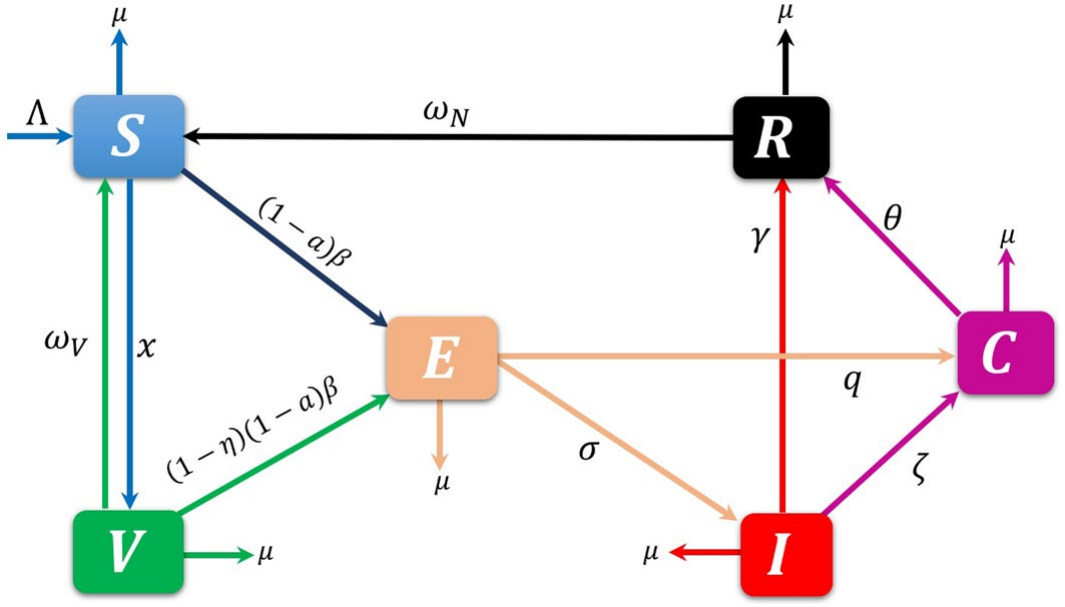
Flow diagram of the epidemiology model (1). The state variables and parameters of the model are described in Table 1.

An accurate mathematical model is essential for gaining insight into disease patterns and devising strategies to manage disease transmission. Various mathematical models using classical integer-order derivatives were proposed to study the disease transmission dynamics and other variables over many years. This work aims to construct a mathematical model for the transmission dynamics of the disease. Let $N(t)$ be the whole population at time $t$. The population comprises mutually exclusive compartments for susceptible $S(t)$, vaccinated $V(t)$, exposed $E(t)$, infected $I(t)$, quarantined, or isolated $C(t)$ and recovered $R(t)$ individuals, which is illustrated in Fig. 1. Thus,

**Table 1:** List of parameters, variables, and their meanings.

Notation	Meaning	Values	References
$\beta$	Transmission rate	0.8333	^3^
$\sigma$	Incubation period	4 days^−1^	Assumed
$x$	Vaccination rate	0.01	^3^
$\gamma$	Recovery rate	10 days^−1^	Assumed
$\Lambda$	Natural birth rate of humans	0.0	Assumed
$\mu$	Natural death rate of humans	0.0	Assumed
${\omega }_{V}$	Vaccination immunity wanes rate	0.0–0.1	Assumed
${\omega }_{N}$	Natural immunity wanes rate	0.0–0.1	Assumed
$q$	Quarantine rate	0.0–0.2	Assumed
$\zeta$	Isolation rate	0.0–0.2	Assumed
$\theta$	Quarantine or isolation period	14 days^−1^	Assumed
$m$	Vaccine uptake	0.1	^3^
${C}_{i}$	Infection cost for individuals	1.0	^3^
${C}_{V}$	Cost for vaccination	0.1, 0.9	Assumed
$S(t)$	Number of susceptible individuals	0.9999	^3^
$V(t)$	Number of vaccinated individuals	0.0	Assumed
$E(t)$	Number of exposed individuals	0.0	^36^
$I(t)$	Number of infected individuals	0.0001	^3^
$C(t)$	Number of quarantined or isolated individuals	0.0	Assumed
$R(t)$	Number of recovered individuals	0.0	^3^

$$\dot{S}\left(t\right)=\Lambda -\left(1-a\right)\beta SI-xS+{\omega }_{V}V+{\omega }_{N}R-\mu S,$$

$$\dot{V}\left(t\right)=xS-\left(1-a\right)(1-\eta )\beta VI-({\omega }_{V}+\mu )V,$$

1 $$\dot{E}\left(t\right)=\left(1-a\right)\beta SI+\left(1-a\right)\left(1-\eta \right)\beta VI-\left(\sigma +q+\mu \right)E,$$

$$\dot{I}\left(t\right)=\sigma E-\left(\gamma +\zeta +\mu \right)I,$$

$$\dot{C}\left(t\right)=qE+\zeta I-\left(\theta +\mu \right)C,$$

$$\dot{R}\left(t\right)=\gamma I+\theta C-({\omega }_{N}+\mu )R.$$

2 $$\mathrm{The total population}, N\left(t\right)=S\left(t\right)+V\left(t\right)+E\left(t\right)+I\left(t\right)+C\left(t\right)+R\left(t\right).$$

Here, the first equation in the model illustrates the dynamics of susceptible people $S(t)$ produced by recruitment through birth and immigration, natural and vaccination immunity waning rate $\Lambda ,{\omega }_{N}, {\text{and}} {\omega }_{V}$. The population in this class decreases when people move to the vaccinated class $V(t)$ and exposed class $E(t)$ at vaccination rate $x$ and transmission rate $\beta$ (person^−1^ day^−1^) after interacting with infectious individuals in $I(t)$. The mortality rate in all classes is $\mu$. Susceptible and vaccinated people are protected from infection by awareness rate $a$. Vaccinated people decreased due to a lack of vaccine efficacy $\eta$ and lack of awareness and entered the exposed class. Exposed people entered the infected class after the incubation period $1/\sigma$, and a specific portion joined the class $C(t)$ through quarantine rate $q$. Infected individuals joined the class $C(t)$ through isolation rate $\zeta$ and recovered class through recovery rate $\gamma$. Quarantine and isolated people joined the recovered class after quarantine or isolation period $1/\theta$. All the state variables and parameters’ biological meanings and values are listed in Table 1.

### Behavior Model

We present a behavior model^27–29^ that describes the continuous change from a state of susceptibility $(S)$ to a state of vaccinated $(V)$, represented by $x$. Here are the behavioral dynamical equations that we define:

3 $$\dot{x}=mx\left(1-x\right)\left(-{C}_{V}V+{C}_{i}I+A\right),$$

where, $m$ denotes the individual’s effort rate. ${C}_{V} and {C}_{i} (=1)$ denotes the infection cost per person and the vaccination cost, respectively. A represents the government forces to participate in vaccination programs. We examine strategy switching by using the concepts of behavioral dynamics in evolutionary game theory, utilizing the formula [$-{C}_{V}V+{C}_{i}I+A$]. The sign of this phrase is crucial in deciding if the recommended action is to participate in an intervention game like vaccination. Let $\Delta P$ represent the difference in payoffs between the two strategies: ${P}_{V}$ for vaccination and ${P}_{I}$ for infected individuals. To evaluate the attractiveness of various techniques, we give an expected payout to each one as described: the equation is ${P}_{I}= I(t) + A$, with ${C}_{i} = 1$, and ${P}_{V}=-V\left(t\right){C}_{V}$.

### Preliminaries and basic properties of fractional calculus

We provide concise explanations, fundamental principles, and propositions related to the Atangana–Baleanu fractional derivative and integral. This specific fractional derivative and integral has become a valuable advancement for academics due to its non-local nature and ability to provide intriguing solutions to real-world situations. Using the Laplace transform is advantageous for solving real-life situations involving initial conditions.

#### Definition 1

The prominent Caputo fractional-order derivative^30^ of a function $H(t)$ with a positive fractional-order $\alpha >0$ can be expressed in the following manner:

$${{}_{0}{}^{C}D}_{t}^{\alpha }H\left(t\right)=\frac{1}{\Gamma \left(n-\alpha \right)}{\int }_{0}^{t}\frac{{H}^{\left(\alpha \right)}\left(v\right)}{(t-v{)}^{\alpha -n-1}}dv,t>0,\alpha >0,n-1<\alpha \le n,n\in {\mathbb{N}}.$$

where, $\Gamma$ signifies the notorious Gamma function.

#### Definition 2

The basic definition of the ABC derivative of a function $H(t)$ is as follows, provided that $H(t)$ belongs to the set ${C}^{1} (0,T)$.

$${{}_{0}{}^{ABC}D}_{t}^{\alpha }H(t)=\frac{ABC\left(\alpha \right)}{1-\alpha }{\int }_{0}^{t}\frac{d}{d\vartheta }H(t){\varepsilon }_{\alpha }\left[\frac{-\alpha }{1-\alpha }{\left(t-\vartheta \right)}^{\alpha }\right]d\vartheta .$$

Here, ${\varepsilon }_{\alpha }$ is the Mittag–Leffler function, and more details are described in^30^.

#### Definition 3

Under the presumption that $H(t)$ is a function on the interval $b[0,T]$, we can get the integral that corresponds to it in the ABC sense by using the following formula:

$${{}_{0}{}^{ABC}I}_{t}^{\alpha }H\left(t\right)=\frac{1-\alpha }{ABC\left(\alpha \right)}H\left(t\right)+\frac{\alpha }{ABC\left(\alpha \right)\Gamma \left(\alpha \right)}{\int }_{0}^{t}{\left(t-\vartheta \right)}^{\alpha -1}H\left(\vartheta \right)d\vartheta .$$

#### Definition 4

^31^ Laplace transformation of the Atangana-Baleanu fractional derivative of fractional-order $\alpha >0$ in the sense of Caputo is as follows, where the lower limit is equal to zero:

$$\mathcal{L}\left({{}_{0}{}^{ABC}D}_{t}^{\alpha }u\left(t\right)\right)=\frac{{B}^{\alpha }u\left(B\right)-{B}^{\alpha -1}u(0)}{{B}^{\alpha }\left(1-\alpha \right)+\alpha }.$$

Now, the fractional-order representation of the model (1.1) is

$${}_{0}{}^{ABC}{D}_{t}^{\alpha }S=\Lambda -\left(1-a\right)\beta SI-xS+{\omega }_{V}V+{\omega }_{N}R-\mu S,$$

$${}_{0}{}^{ABC}{D}_{t}^{\alpha }V=xS-\left(1-a\right)\left(1-\eta \right)\beta VI-({\omega }_{V}+\mu )V,$$

4 $${}_{0}{}^{ABC}{D}_{t}^{\alpha }E=\left(1-a\right)\beta SI+\left(1-a\right)\left(1-\eta \right)\beta VI-\left(\sigma +q+\mu \right)E,$$

$${}_{0}{}^{ABC}{D}_{t}^{\alpha }I=\sigma E-\left(\gamma +\zeta +\mu \right)I,$$

$${}_{0}{}^{ABC}{D}_{t}^{\alpha }C=qE+\zeta I-\left(\theta +\mu \right)C,$$

$${}_{0}{}^{ABC}{D}_{t}^{\alpha }R=\gamma I+\theta C-({\omega }_{N}+\mu )R.$$

It is assumed that every biological parameter incorporated in the model, as mentioned earlier, is positive. In the proposed model (4), the fractional derivative ${}_{0}{}^{ABC}{D}_{t}^{\alpha }$ is treated as the Atangana-Baleanu derivative in the Caputo sense (ABC), as opposed to the traditional integer-order derivative. The rationale behind this is that classical integer-order models lack the memory effects present in numerous biological models.

## Mathematical analysis

### Positivity of the model solution

Investigating the positivity of dynamical system solutions is a great way to ensure the initial conditions are not negative. This study appears in various mathematical and epidemiological modeling literature.

#### Lemma 1

*If the initial conditions are non-negative and will also reside in* ${\mathbb{R}}_{+}^{6}$, *then the fractional order model (4) solution is non-negative*.

#### Proof

The vector field points must be in ${\mathbb{R}}_{+}^{6}$ on each hyperplane boundary to prove the values are positive. As a result, according to the considered fractional order model, one gets,

$${}_{0}{}^{ABC}{D}_{t}^{\alpha }S{\left. {\vphantom{0}}\right|}_{S=0}=\Lambda +{\omega }_{V}V+{\omega }_{N}R\ge 0,$$

$${}_{0}{}^{ABC}{D}_{t}^{\alpha }V{\left.{\vphantom{0}}\right|}_{V=0}=xS\ge 0,$$

$${}_{0}{}^{ABC}{D}_{t}^{\alpha }E{\left.{\vphantom{0}}\right|}_{E=0}=\left(1-a\right)\beta SI+\left(1-a\right)\left(1-\eta \right)\beta VI\ge 0,$$

$${}_{0}{}^{ABC}{D}_{t}^{\alpha }I{\left.{\vphantom{0}}\right|}_{I=0}=\sigma E\ge0 ,$$

$${}_{0}{}^{ABC}{D}_{t}^{\alpha }C{\left.{\vphantom{0}}\right|}_{C=0}=qE+\zeta I\ge 0,$$

$${}_{0}{}^{ABC}{D}_{t}^{\alpha }R{\left.{\vphantom{0}}\right|}_{R=0}=\gamma I+\theta C\ge 0.$$

Therefore, according to Ref.^31^, it is evident that the solution of the proposed fractional-order model (4) is positive.

The above proof guarantees that if the initial values of the model variables are non-negative, then the variable values at the model solution will always be non-negative throughout time. This result is significant since the variables involved are human populations that typically do not have negative values.

### Models invariant region

In this section, using the following lemma, we study the region where the solutions of the fractional-order non-linear system (4) are positively bounded and mathematically well-posed, more precisely, the validity of the proposed model’s solutions.

#### Lemma 2

*The fractional-order model (4) does not negatively affect the closed set*

$$\Omega =\left\{\left(S,V,E,I,C,R\right)\in {\mathbb{R}}^{6}:0\le S+V+E+I+C+R\le \frac{\Lambda }{\mu }\right\}.$$

#### Proof

The fractional-order derivative of the total population can be calculated as

$${}_{0}{}^{ABC}{D}_{t}^{\alpha }\left(S+V+E+I+C+R\right)=\Lambda -\upmu \left(S+V+E+I+C+R\right),$$

5 $$\Rightarrow {}_{0}{}^{ABC}{D}_{t}^{\alpha }N\le\Lambda -\mathrm{\mu N}.$$

According to the Laplace transform formula, one can write Eq. (5) as follows:

6 $$N\left(t\right)\le \left(\frac{B\left(\alpha \right)}{B\left(\alpha \right)+\left(1-\alpha \right)\mu }N\left(0\right)+\frac{\left(1-\alpha \right)\Lambda }{B\left(\alpha \right)+\left(1-\alpha \right)\mu }\right){E}_{\alpha ,1}\left(-\delta {t}^{\alpha }\right)+\frac{\alpha\Lambda }{B\left(\alpha \right)+\left(1-\alpha \right)\mu }{E}_{\alpha ,\alpha +1}\left(-\delta {t}^{\alpha }\right),$$

$${\text{where}}, \delta =\frac{\alpha \mu }{B\left(\alpha \right)+\left(1-\alpha \right)\mu } {\text{and}} {E}_{*,*} \mathrm{known as two parameters Mittag}-\mathrm{Leffler function}.$$

As reference^32^ stated, the Mittag–Leffler function exhibits an asymptotic behavior. Thus, when $t\to \infty$, we can write $N\left(t\right)\le \frac{\Lambda }{\mu }.$ Hence, the model (4) solution persists in the invariant set $\Omega .$ Therefore, the fractional-order model (4) does not negatively affect the closed set $\Omega$.

From an epidemiological perspective, a model solution is considered bounded if solutions commence near the initial circumstances and stay indefinitely inside a particular closed domain.

### Basic reproduction number

In general, the fundamental reproduction number ${R}_{0}$ of the model represents the number of new infectious cases that result from a solitary typical infection within a population that is entirely susceptible. This section uses the next-generation approach^33^ to determine the model’s basic reproduction number $({R}_{0})$. Solve the system below to get a disease-free $(I=0)$ equilibrium point ${E}_{0}$ of the suggested fractional-order model as follows:

$${}_{0}{}^{ABC}{D}_{t}^{\alpha }S={}_{0}{}^{ABC}{D}_{t}^{\alpha }V={}_{0}{}^{ABC}{D}_{t}^{\alpha }E={}_{0}{}^{ABC}{D}_{t}^{\alpha }I={}_{0}{}^{ABC}{D}_{t}^{\alpha }C={}_{0}{}^{ABC}{D}_{t}^{\alpha }R=0.$$

Thus, one gets

$${E}_{0}=\left(\frac{\Lambda ({\upomega }_{{\text{V}}}+\mu )}{\mu (x+{\upomega }_{{\text{V}}}+\mu )},\frac{\mathrm{x\Lambda }}{\mu (x+{\upomega }_{{\text{V}}}+\mu )},\mathrm{0,0},\mathrm{0,0}\right).$$

According to Ref.^33^, the inflow and outflow matrix is as follows (non-demographic case):

$$F=\left[\begin{array}{ccc}0& \left(1-a\right)\beta +\left(1-a\right)\left(1-\eta \right)\beta & 0\\ 0& 0& 0\\ 0& 0& 0\end{array}\right],$$ and $$V=\left[\begin{array}{ccc}\sigma +q& 0& 0\\ -\sigma & \gamma +\zeta & 0\\ -q& -\zeta & \theta \end{array}\right].$$

In the sense of the spectral radius of the matrix $F{V}^{-1}$, the proposed model desired ${R}_{0}$ is

$${R}_{0}=\frac{\sigma [\left(1-a\right)\beta +\left(1-a\right)\left(1-\eta \right)\beta ]}{(\sigma +q)(\gamma +\zeta )}.$$

### Existence and uniqueness of the solution

This section will explore the existence and uniqueness of the solution for model (4). We use Krasnoselskii’s fixed point theory for this purpose, as the existence and uniqueness of a solution are crucial characteristics of differential equations. Many academics have examined the phenomena of differential equations containing fractional order derivatives and integrals^34,35^. We rewrite model (4) in a simplified version as follows:

7 $$\left\{\begin{array}{c}{}_{0}{}^{ABC}{D}_{t}^{\alpha }u\left(t\right)=H\left(t,u\left(t\right)\right),\\ u\left(0\right)={u}_{0}, 0<t<T<\infty .\end{array}\right.$$

System (4) represents the system with $u(t)$ as a vector consisting of state variables $(S, V, E, I, C, R)$, where $H$ is a continuous vector function. Thus

$$H=\left(\begin{array}{c}{H}_{1}\\ {H}_{2}\\ {H}_{3}\\ {H}_{4}\\ {H}_{5}\\ {H}_{6}\end{array}\right)=\left(\begin{array}{c}\Lambda -\left(1-a\right)\beta SI-xS+{\omega }_{V}V+{\omega }_{N}R-\mu S\\ xS-\left(1-a\right)\left(1-\eta \right)\beta VI-({\omega }_{V}+\mu )V\\ \left(1-a\right)\beta SI+\left(1-a\right)\left(1-\eta \right)\beta VI-\left(\sigma +q+\mu \right)E\\ \sigma E-\left(\gamma +\zeta +\mu \right)I\\ qE+\zeta I-\left(\theta +\mu \right)C\\ \gamma I+\theta C-({\omega }_{N}+\mu )R\end{array}\right),$$

where, the initial condition of the state variables is ${u}_{0}\left(t\right)=(S\left(0\right),V\left(0\right),E\left(0\right),I\left(0\right),C\left(0\right),R\left(0\right)).$ According to Lipschitz condition, one can write,

8 $$\Vert H(t,{u}_{1}\left(t\right))-H(t,{u}_{2}\left(t\right))\Vert \le M\Vert {u}_{1}\left(t\right)-{u}_{2}\left(t\right)\Vert .$$

Now, we offer the following theorem to verify the existence and uniqueness of the system solution (4).

#### Theorem

*The following condition ensures a unique solution for the fractional-order model (4).*

9 $$\frac{1-\alpha }{ABC\left(\alpha \right)}{\mathbb{Q}}+\frac{\alpha }{\Gamma \left(\alpha \right)ABC\left(\alpha \right)}{\mathbb{Q}}{T}_{max}^{\alpha }<1$$

#### Proof

Equation (7) can be written as

10 $$u\left(t\right)-u\left(0\right)=\frac{1-\alpha }{{\mathbb{A}}{\mathbb{B}}{\mathbb{C}}\left(\alpha \right)}H\left(t,u\left(t\right)\right)+\frac{\alpha }{\Gamma \left(\alpha \right){\mathbb{A}}{\mathbb{B}}{\mathbb{C}}\left(\alpha \right)}{\int }_{0}^{t}{\left(t-\vartheta \right)}^{\alpha -1}H\left(\vartheta ,u\left(\vartheta \right)\right)d\vartheta .$$

Assume $b=(0,T)$; according to the operator $\Theta :\complement (b,{\mathbb{R}}^{6})\to \complement (b,{\mathbb{R}}^{6})$, one can write,

11 $$\Theta \left[u\left(t\right)\right]={u}_{0}+\frac{1-\alpha }{{\mathbb{A}}{\mathbb{B}}{\mathbb{C}}\left(\alpha \right)}H\left(t,u\left(t\right)\right)+\frac{\alpha }{\Gamma \left(\alpha \right){\mathbb{A}}{\mathbb{B}}{\mathbb{C}}\left(\alpha \right)}{\int }_{0}^{t}{\left(t-\vartheta \right)}^{\alpha -1}H\left(\vartheta ,u\left(\vartheta \right)\right)d\vartheta .$$

12 $$\therefore \left(10\right)\Rightarrow u\left(t\right)=\Theta \left[u\left(t\right)\right].$$

Thus, on set $b$ the norm of supremum$,{\Vert .\Vert }_{b}$ is

13 $${\Vert u\left(t\right)\Vert }_{b}=\underset{t\epsilon b}{{\text{sup}}}\Vert u(t)\Vert , u\left(t\right)\in \complement .$$

Sufficient evidence to form a Banach space.

In addition, the following inequality can be shown with ease:

14 $$\Vert {\int }_{0}^{t}G\left(t,\vartheta \right)u\left(\vartheta \right)d\vartheta \Vert \le T{\Vert G\left(t,\vartheta \right)\Vert }_{b}{\Vert u\left(t\right)\Vert }_{b},$$

where, $$u\left(t\right)\in \complement \left(b,{\mathbb{R}}^{6}\right), G\left(t,\vartheta \right)\in \complement \left({b}^{2},{\mathbb{R}}^{6}\right).$$

15 $${\text{Therefore}}, {\Vert G\left(t,\vartheta \right)\Vert }_{b}=\underset{t,\vartheta \epsilon b}{{\text{sup}}}\left|G\left(t,\vartheta \right)\right|.$$

Using Eq. (12), one can write,

16 $${\Vert \Theta \left[{u}_{1}\left(t\right)\right]-\Theta \left[{u}_{2}\left(t\right)\right]\Vert }_{b}\le {\Vert \frac{1-\alpha }{{\mathbb{A}}{\mathbb{B}}{\mathbb{C}}\left(\alpha \right)}\left(H\left(t,{u}_{1}\left(t\right)\right)-H\left(t,{u}_{1}\left(t\right)\right)\right)+\frac{\alpha }{\Gamma \left(\alpha \right){\mathbb{A}}{\mathbb{B}}{\mathbb{C}}\left(\alpha \right)}{\int }_{0}^{t}{\left(t-\vartheta \right)}^{\alpha -1}\left(H\left(\vartheta ,{u}_{1}\left(\vartheta \right)\right)-H\left(\vartheta ,{u}_{2}\left(\vartheta \right)\right)\right)d\vartheta \Vert }{}_{b}.$$

Additionally, after simplifying, we got by using the triangle inequality, the Lipschitz condition (8), and the result in (14),

$${\Vert \Theta \left[{u}_{1}\left(t\right)\right]-\Theta \left[{u}_{2}\left(t\right)\right]\Vert }_{b}\le \frac{1-\alpha }{ABC\left(\alpha \right)}{\mathbb{Q}}\Vert {u}_{1}\left(t\right)-{u}_{2}\left(t\right)\Vert {}_{b}+\frac{\alpha }{\Gamma \left(\alpha \right)ABC\left(\alpha \right)}{\mathbb{Q}}{T}_{max}^{\alpha }\Vert {u}_{1}\left(t\right)-{u}_{2}\left(t\right)\Vert {}_{b}.$$

17 $${\Rightarrow \Vert \Theta \left[{u}_{1}\left(t\right)\right]-\Theta \left[{u}_{2}\left(t\right)\right]\Vert }_{b}\le M\Vert {u}_{1}\left(t\right)-{u}_{2}\left(t\right)\Vert {}_{b}.$$

Here, the operator $M=\frac{1-\alpha }{ABC\left(\alpha \right)}{\mathbb{Q}}+\frac{\alpha }{\Gamma \left(\alpha \right)ABC\left(\alpha \right)}{\mathbb{Q}}{T}_{max}^{\alpha }$, is a contraction, and according to Banach’s contraction principle, it has a unique fixed point^31^. This fact completes the proof of the theorem mentioned above.

## Results and discussions

**Figure 2:**
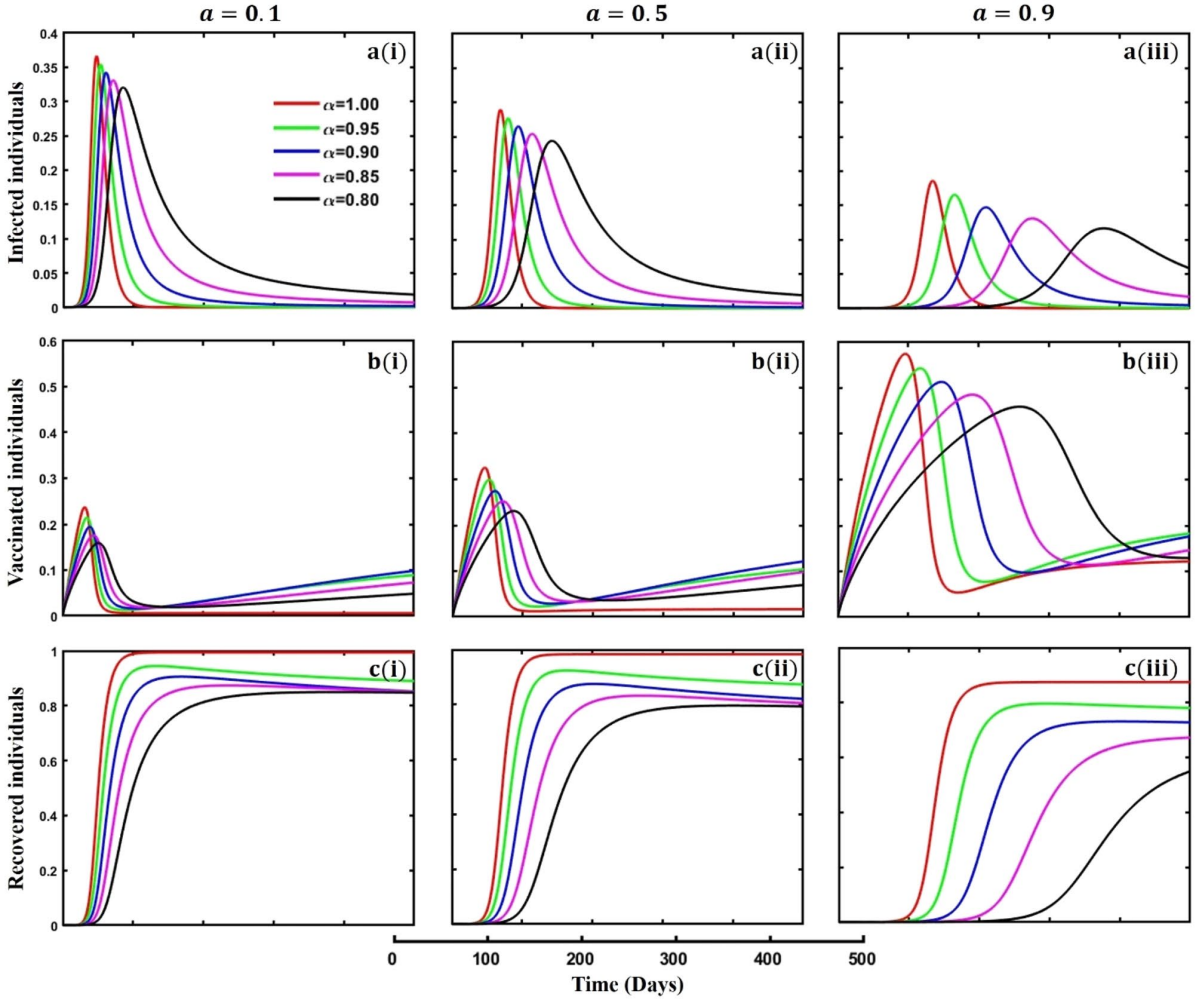
The effect of fractional-order $\alpha =0.8, 0.85, 0.9,\mathrm{ 0.95,1.0}$ on the infected (Panel **a**-*), vaccinated (Panel **b**-*), and recovered (Panel **c**-*) individuals. Subpanels (*-i), (*-ii), and (*-iii) show the results of awareness rate $a=0.1, 0.5$ and $0.9,$ respectively, whereas the remaining parameter settings are $\beta =0.8333, \gamma =0.1, x=0.01,\sigma =1/4,\eta =0.5, q=0.0,\upzeta =0.0,{\omega }_{V}=0.0,{\omega }_{N}=0.0,$ and $\theta =1/14$.

**Figure 3:**
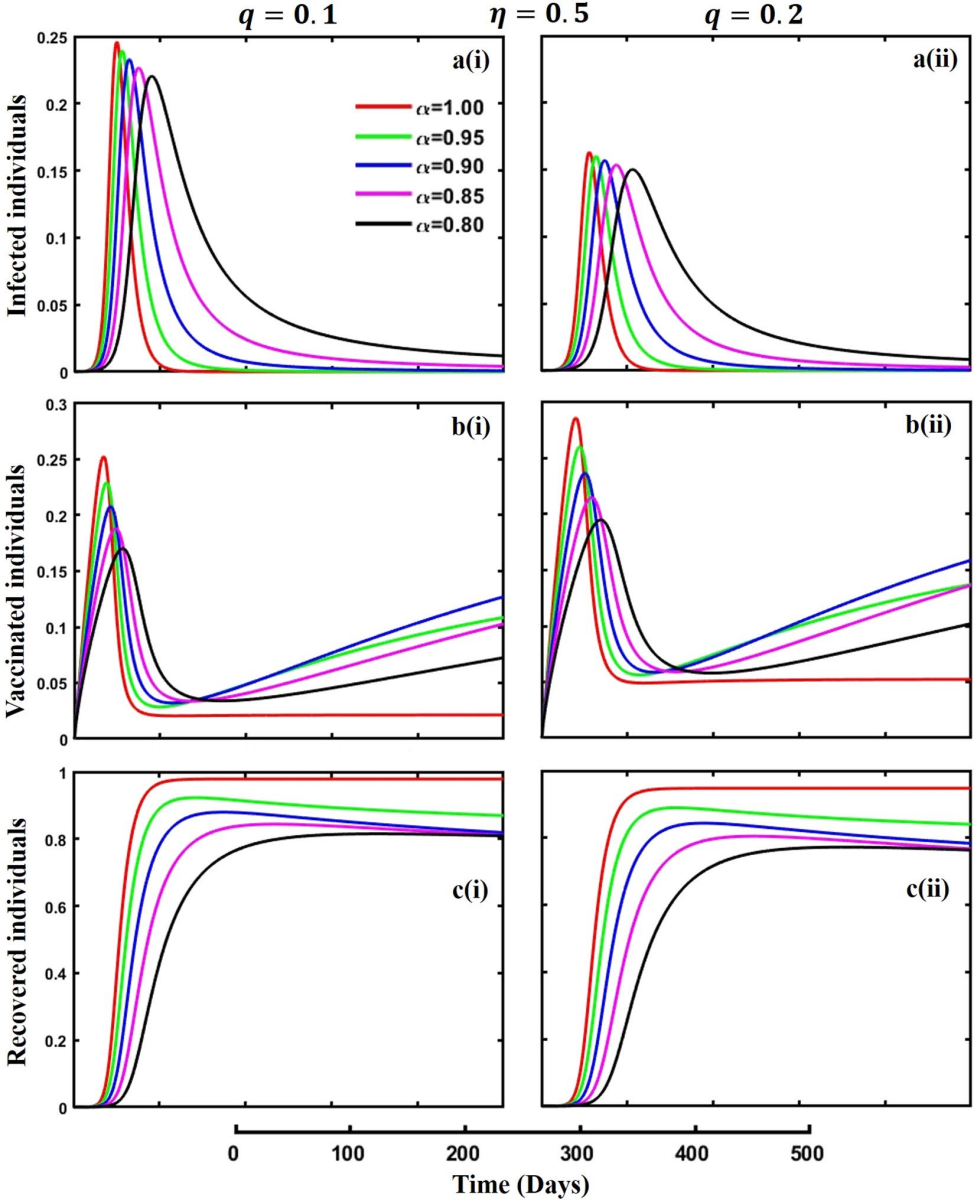
The effect of fractional-order $\alpha =0.8, 0.85, 0.9,\mathrm{ 0.95,1.0}$ on the infected (Panel **a**-*), vaccinated (Panel **b**-*), and recovered (Panel **c**-*) individuals. Subpanels (*-i) and (*-ii) show the results of quarantine rate $q=0.1$ and $0.2,$ respectively, whereas the remaining parameter settings are $\beta =0.8333, \gamma =0.1, x=0.01,\sigma =1/4,\eta =0.5,\upzeta =0.0,{\omega }_{V}=0.0,{\omega }_{N}=0.0,a=0.0$ and $\theta =1/14$.

**Figure 4:**
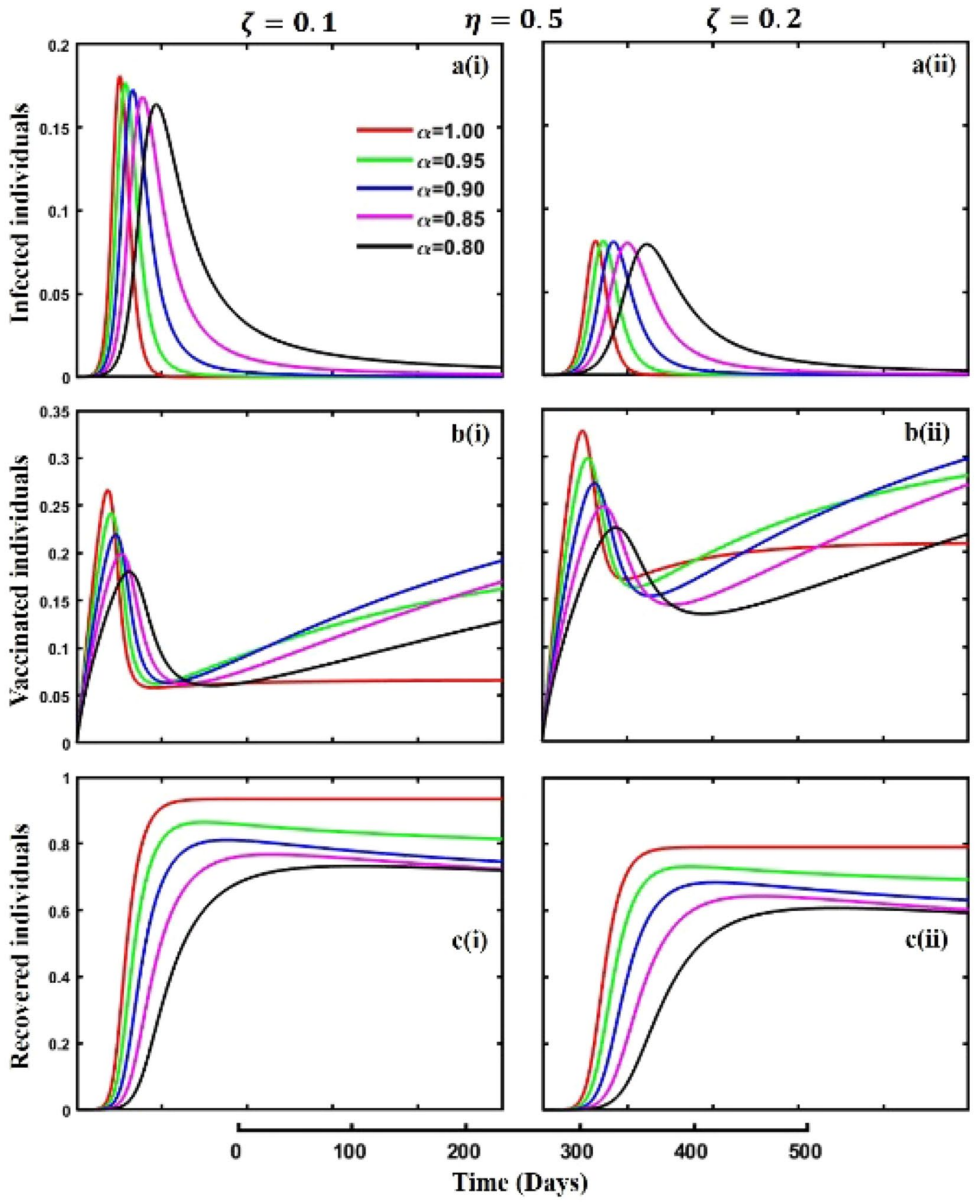
The effect of fractional-order $\alpha =0.8, 0.85, 0.9,\mathrm{ 0.95,1.0}$ on the infected (Panel **a**-*), vaccinated (Panel **b**-*), and recovered (Panel **c**-*) individuals. Subpanels (*-i) and (*-ii) show the results of isolation rate $\upzeta =0.1$ and $0.2,$ respectively, whereas the remaining parameter settings are $\beta =0.8333, \gamma =0.1, x=0.01,\sigma =1/4,\eta =0.5, q=0.0,{\omega }_{V}=0.0,{\omega }_{N}=0.0,a=0.0$ and $\theta =1/14$.

**Figure 5:**
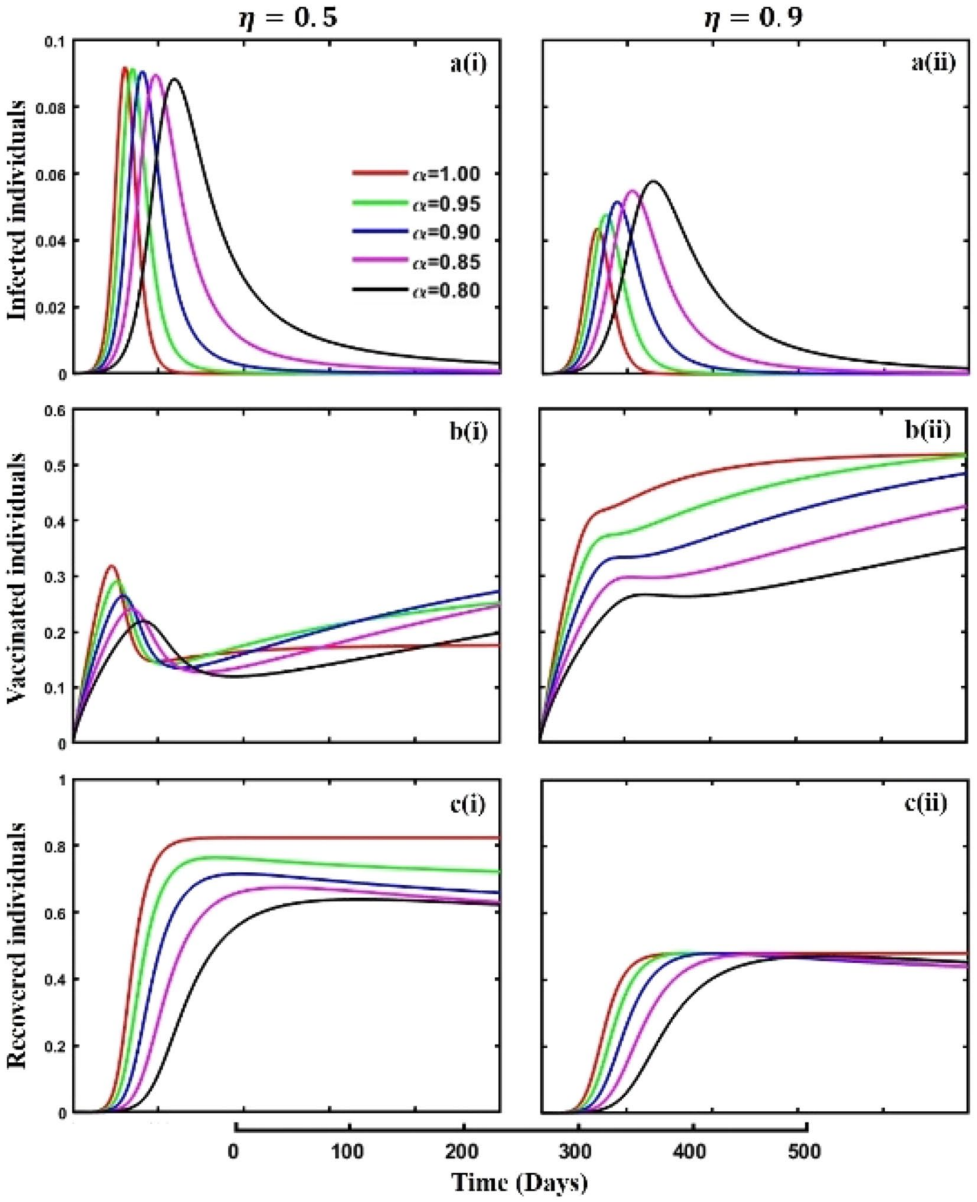
The effect of fractional-order $\alpha =0.8, 0.85, 0.9,\mathrm{ 0.95,1.0}$ on the infected (Panel **a**-*), vaccinated (Panel **b**-*), and recovered (Panel **c**-*) individuals. Subpanels (*-i) and (*-ii) shows the outcome of vaccine efficacy $\eta =0.5$ and $0.9$, respectively, whereas, remaining parameter settings are $\beta =0.8333, \gamma =0.1, x=0.01,\sigma =1/4, q=\upzeta =0.1,{\omega }_{V}=0.0,{\omega }_{N}=0.0,a=0.0$ and $\theta =1/14$.

**Figure 6:**
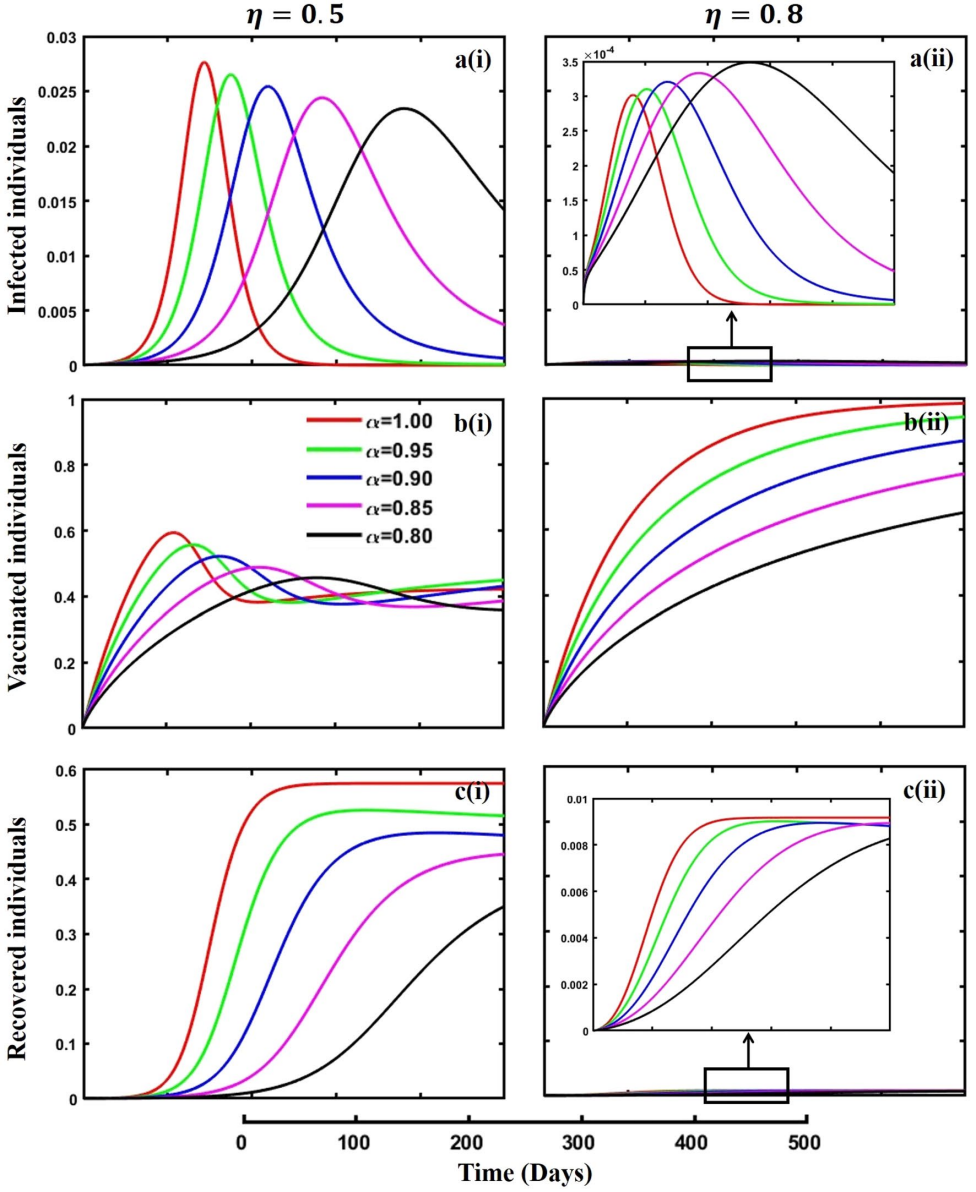
The effect of fractional-order $\alpha =0.8, 0.85, 0.9,\mathrm{ 0.95,1.0}$ on the infected (Panel **a**-*), vaccinated (Panel **b**-*), and recovered (Panel **c**-*) individuals. Subpanels (*-i) and (*-ii) shows the results of vaccine efficacy $\eta =0.5$ and $0.8$, respectively, whereas, remaining parameter settings are $\beta =0.8333, \gamma =0.1, x=0.01,\sigma =1/4,\mathrm{q }=\upzeta =0.1,{\omega }_{V}=0.0,{\omega }_{N}=0.0,a=0.5$ and $\theta =1/14$.

**Figure 7:**
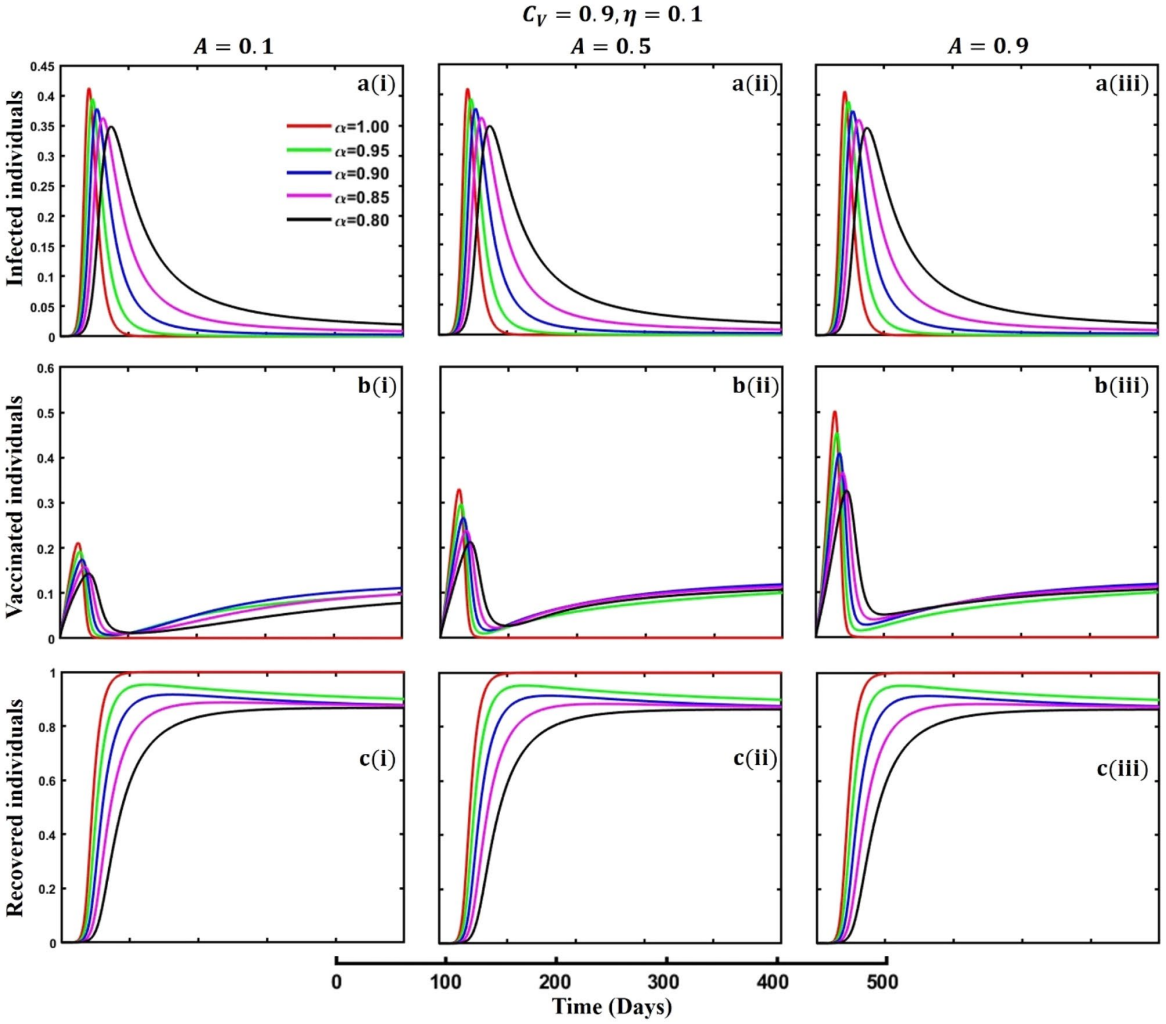
The effect of fractional-order $\alpha =0.8, 0.85, 0.9,\mathrm{ 0.95,1.0}$ on the infected (Panel **a**-*), vaccinated (Panel **b**-*), and recovered (Panel **c**-*) individuals. Subpanels (*-i), (*-ii), and (*-iii) shows the results of government force to participate in vaccination program $A=\mathrm{0.1,0.5}$ and $0.9,$ respectively, whereas the remaining parameter settings are $\beta =0.8333, \gamma =0.1, x=0.01,\sigma =1/4,\eta =0.1, {C}_{V}=0.9,q=\upzeta =0.0,{\omega }_{V}=0.0,{\omega }_{N}=0.0,a=0.0,m=0.1$ and $\theta =1/14$.

**Figure 8:**
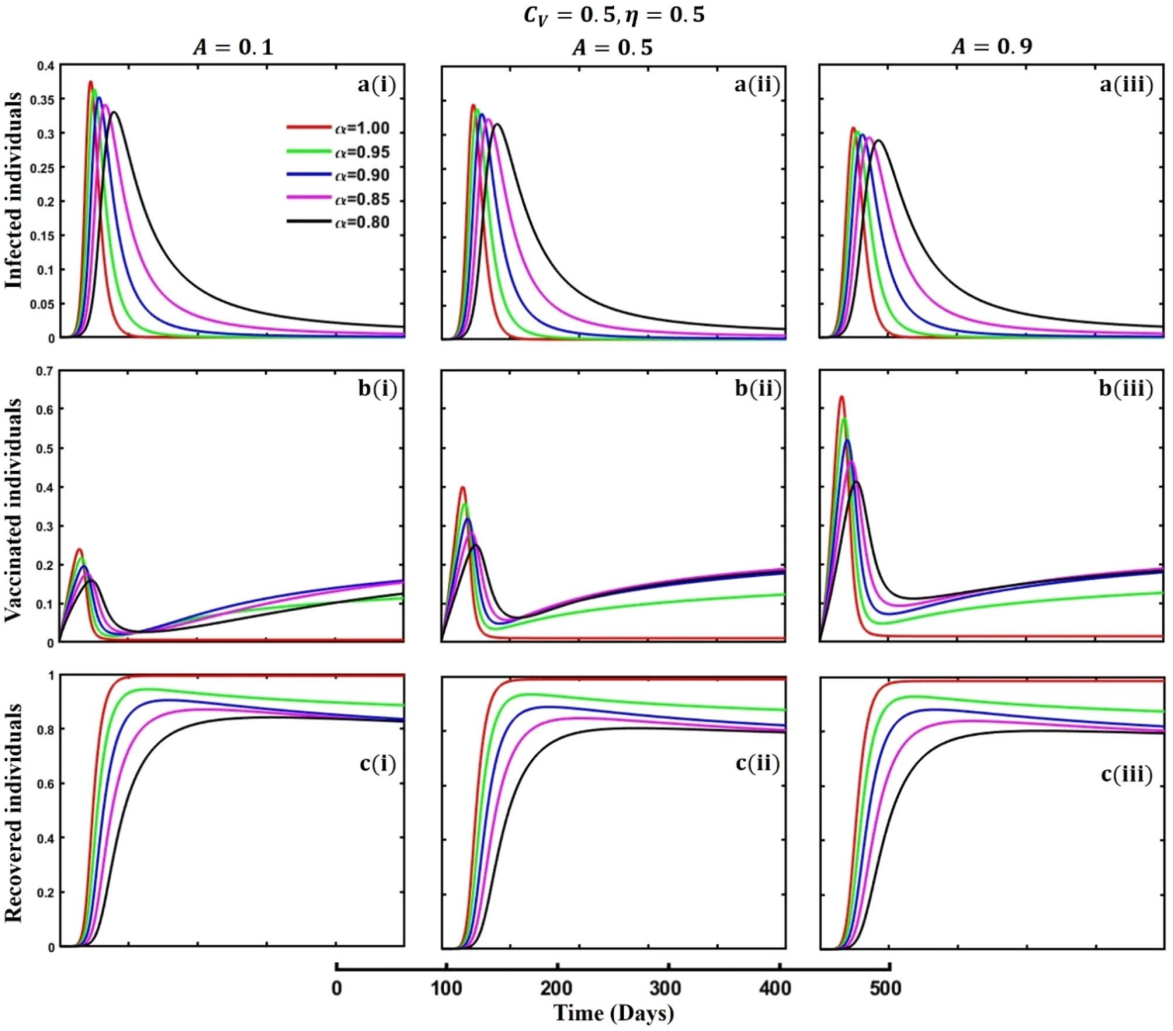
The effect of fractional-order $\alpha =0.8, 0.85, 0.9,\mathrm{ 0.95,1.0}$ on the infected (Panel **a**-*), vaccinated (Panel **b**-*), and recovered (Panel **c**-*) individuals. Subpanels (*-i), (*-ii), and (*-iii) shows the results of government force to participate in vaccination program $A=\mathrm{0.1,0.5}$ and $0.9,$ respectively, whereas the remaining parameter settings are $\beta =0.8333, \gamma =0.1, x=0.01,\sigma =1/4,\eta =0.5, {C}_{V}=0.5,q=\upzeta =0.0,{\omega }_{V}=0.0,{\omega }_{N}=0.0,a=0.0,m=0.1$ and $\theta =1/14$.

**Figure 9:**
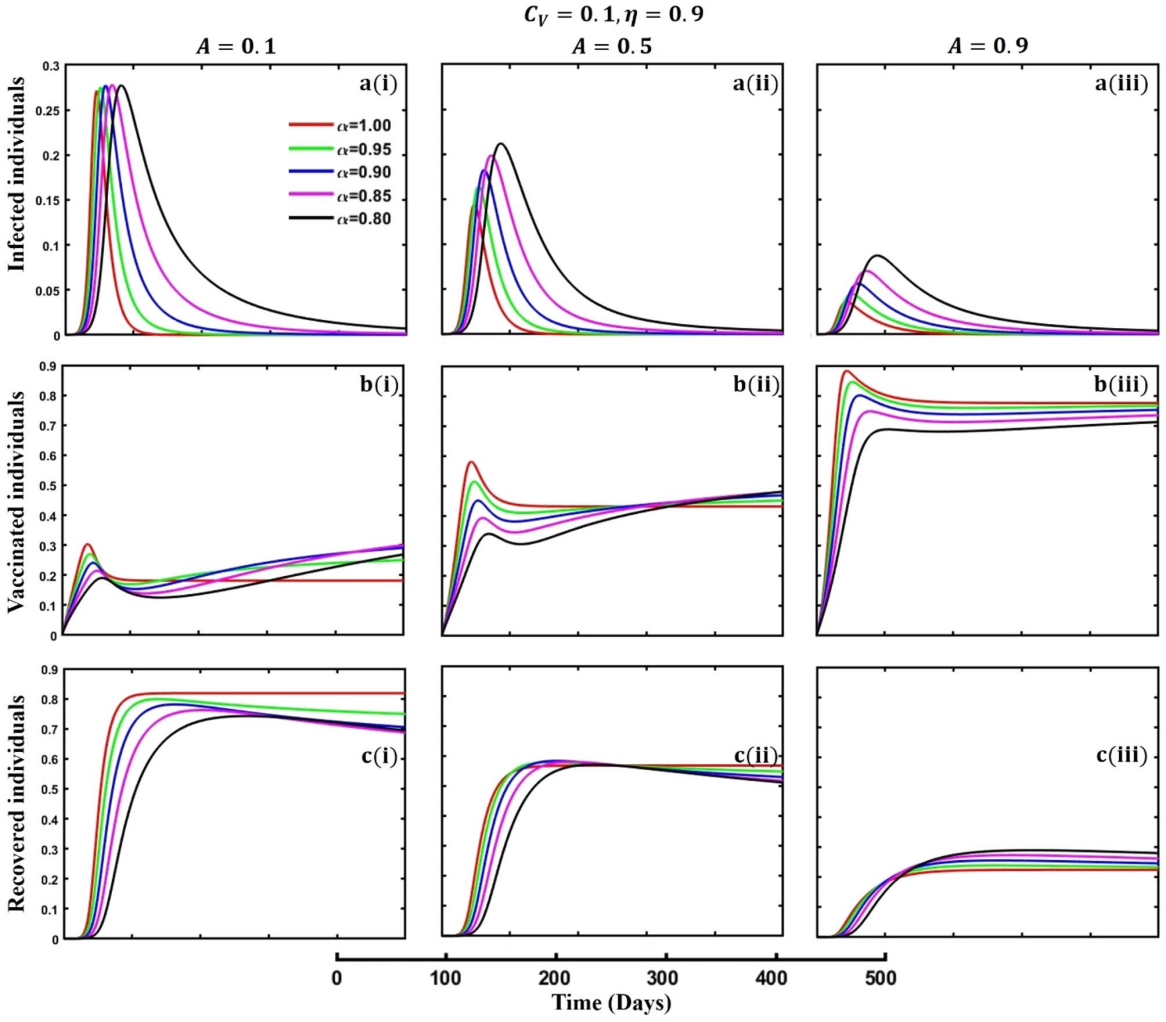
The effect of fractional-order $\alpha =0.8, 0.85, 0.9,\mathrm{ 0.95,1.0}$ on the infected (Panel **a**-*), vaccinated (Panel **b**-*), and recovered (Panel **c**-*) individuals. Subpanels (*-i), (*-ii), and (*-iii) shows the results of government force to participate in vaccination program $A=\mathrm{0.1,0.5}$ and $0.9,$ respectively, whereas the remaining parameter settings are $\beta =0.8333, \gamma =0.1, x=0.01,\sigma =1/4,\eta =0.9, {C}_{V}=0.1,q=\upzeta =0.0,{\omega }_{V}=0.0,{\omega }_{N}=0.0,a=0.0,m=0.1$ and $\theta =1/14$.

**Figure 10:**
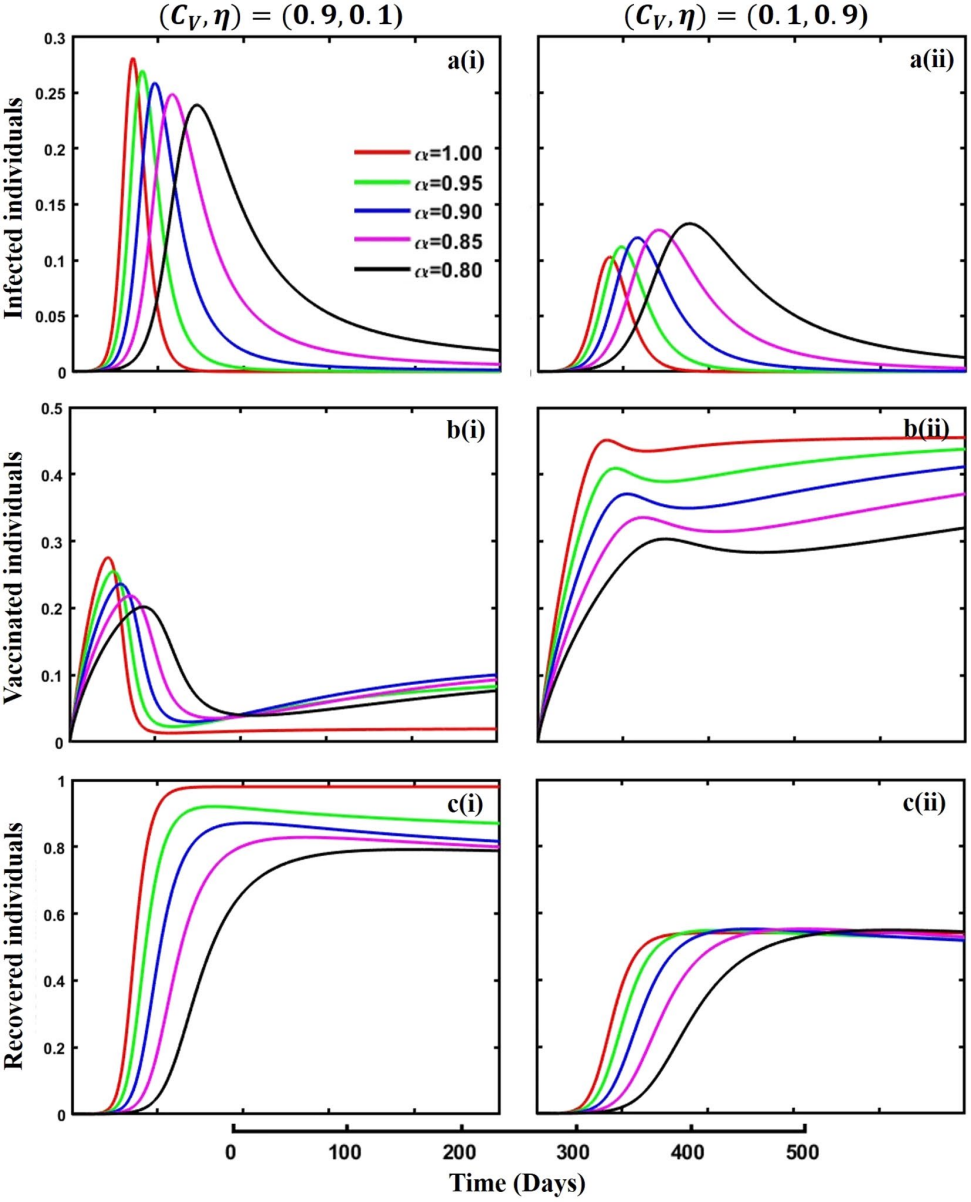
The effect of fractional-order $\alpha =0.8, 0.85, 0.9,\mathrm{ 0.95,1.0}$ on the infected (Panel **a**-*), vaccinated (Panel **b**-*), and recovered (Panel **c**-*) individuals. Subpanels (*-i) and (*-ii) show the results of vaccination cost and efficacy $({C}_{V},\eta )=(\mathrm{0,9},0.1)$ and $(\mathrm{0,1},0.9)$ respectively, whereas, the remaining parameter settings are $\beta =0.8333, \gamma =0.1, x=0.01,\sigma =0.25, q=\upzeta =0.0,{\omega }_{V}=0.0,{\omega }_{N}=0.0,a=0.5,A=0.0,m=0.1$ and $\theta =1/14$.

**Figure 11:**
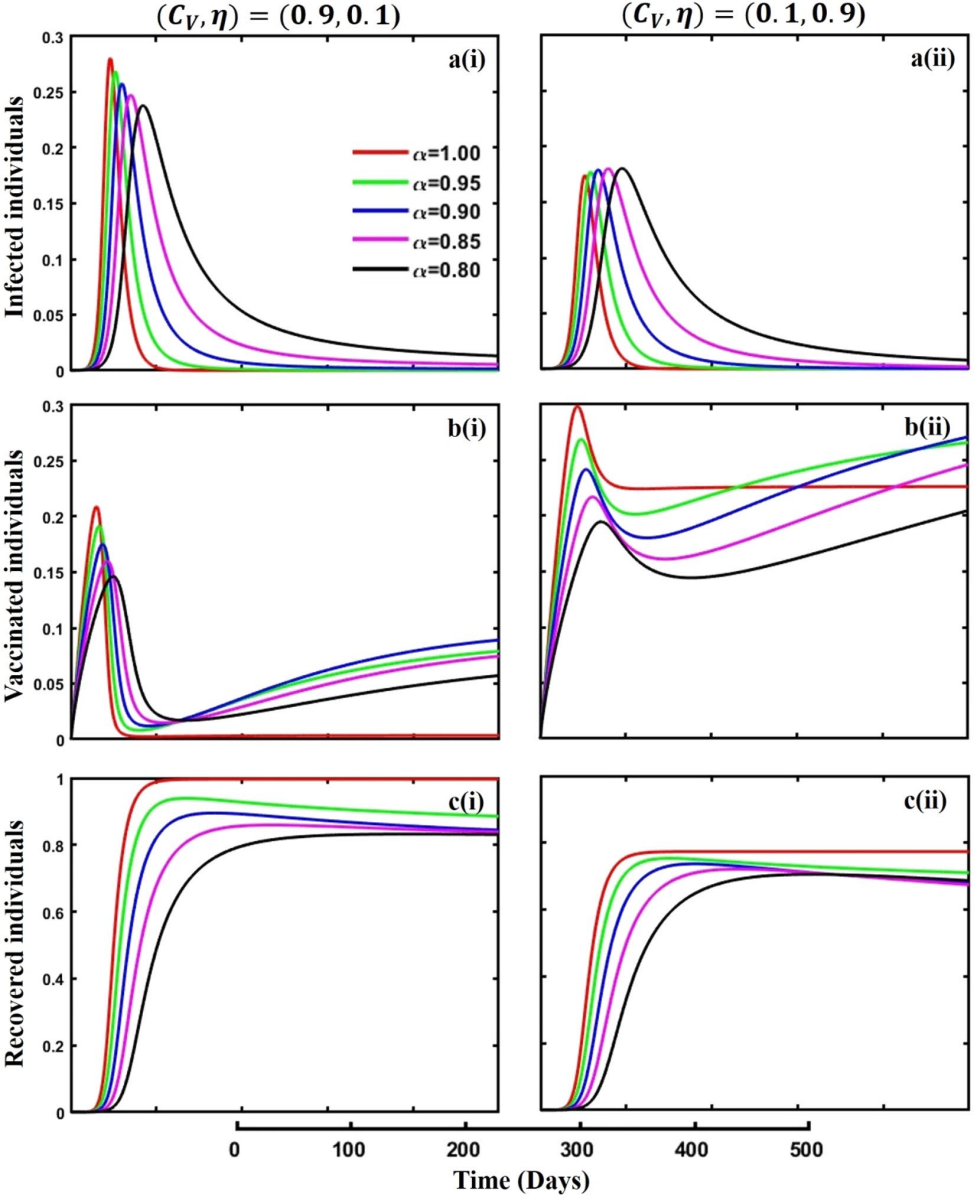
The effect of fractional-order $\alpha =0.8, 0.85, 0.9,\mathrm{ 0.95,1.0}$ on the infected (Panel **a**-*), vaccinated (Panel **b**-*), and recovered (Panel **c**-*) individuals. Subpanels (*-i) and (*-ii) show the outcomes of vaccination cost and efficacy $({C}_{V},\eta )=(\mathrm{0,9},0.1)$ and $(\mathrm{0,1},0.9)$ respectively, whereas, the remaining parameter settings are $\beta =0.8333, \gamma =0.1, x=0.01,\sigma =0.25, q=0.1,\upzeta =0.0,{\omega }_{V}=0.0,{\omega }_{N}=0.0,a=0.0,A=0.0,m=0.1$ and $\theta =1/14$.

**Figure 12:**
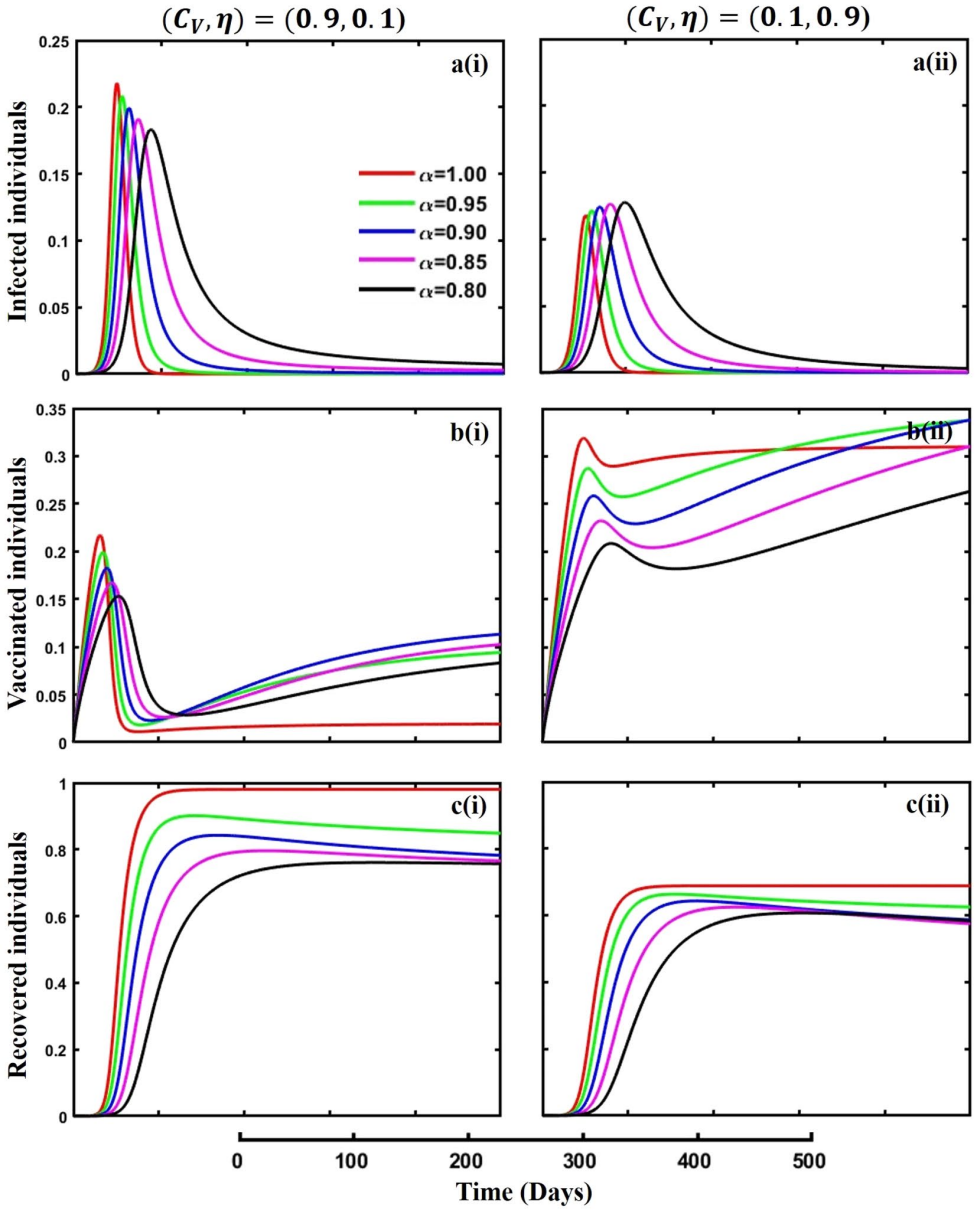
The effect of fractional-order $\alpha =0.8, 0.85, 0.9,\mathrm{ 0.95,1.0}$ on the infected (Panel **a**-*), vaccinated (Panel **b**-*), and recovered (Panel **c**-*) individuals. Subpanels (*-i) and (*-ii) show the results of vaccination cost and efficacy $({C}_{V},\eta )=(\mathrm{0,9},0.1)$ and $(\mathrm{0,1},0.9)$ respectively, whereas, remaining parameter settings are $\beta =0.8333, \gamma =0.1, x=0.01,\sigma =0.25, , q=0.0,\upzeta =0.1,{\omega }_{V}=0.0,{\omega }_{N}=0.0,a=0.0,A=0.0,m=0.1$ and $\theta =1/14$.

**Figure 13:**
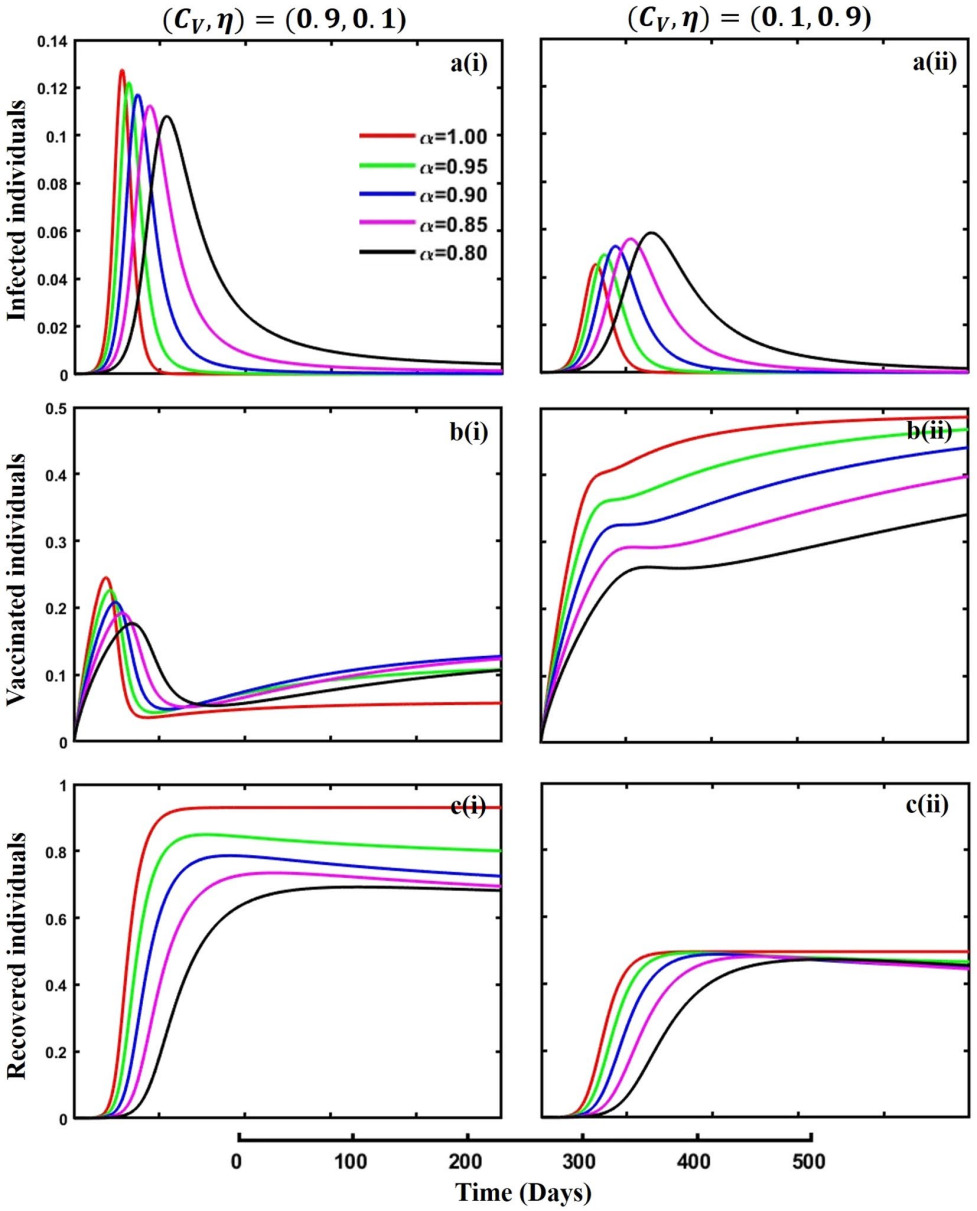
The effect of fractional-order $\alpha =0.8, 0.85, 0.9,\mathrm{ 0.95,1.0}$ on the infected (Panel **a**-*), vaccinated (Panel **b**-*), and recovered (Panel **c**-*) individuals. Subpanels (*-i) and (*-ii) show the results under the vaccination cost and efficacy $({C}_{V},\eta )=(\mathrm{0,9},0.1)$ and $(\mathrm{0,1},0.9)$ respectively, whereas, the remaining parameter settings are $\beta =0.8333, \gamma =0.1, x=0.01,\sigma =0.25, q=0.1,\upzeta =0.1,{\omega }_{V}=0.0,{\omega }_{N}=0.0,a=0.0,m=0.1,A=0.0$ and $\theta =1/14$.

**Figure 14:**
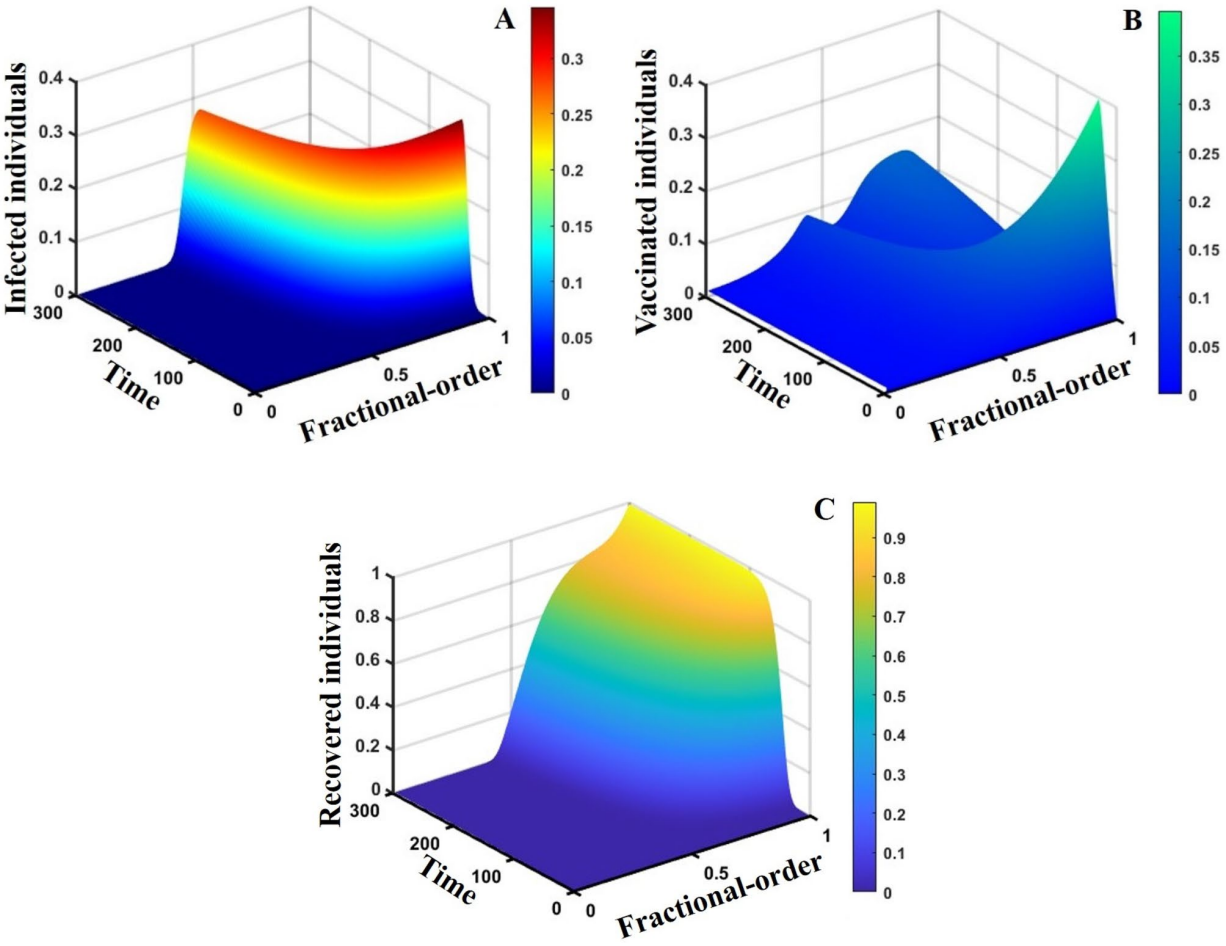
3D surface showing the effect of fractional order $(0\le \alpha \le 1)$ and time elapsed on the (**A**) infected, (**B**) vaccinated, and (**C**) recovered population, where parameters used are $\beta =0.8333, \gamma =0.1,\sigma =1/4,\eta =0.5, q=0.0,\upzeta =0.0,{\omega }_{V}=0.0,{\omega }_{N}=0.0,m=0.1,{C}_{V}=0.5,\eta =0.5,A=0.5,a=0.0$ and $\theta =1/14$.

**Figure 15:**
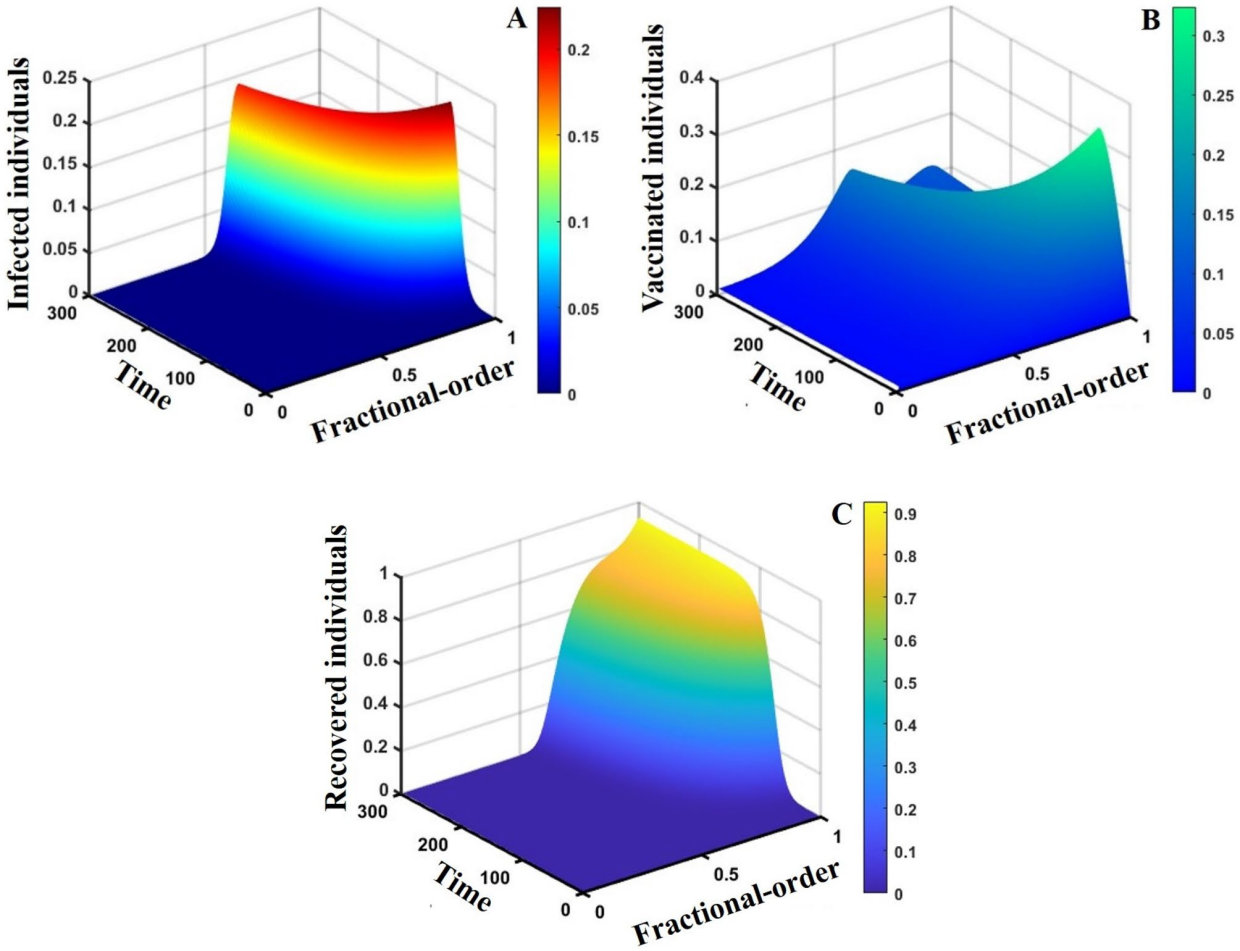
3D surface showing the effect of fractional order $(0\le \alpha \le 1)$ and time elapsed on the (**A**) infected, (**B**) vaccinated, and (**C**) recovered population, where parameters used are $\beta =0.8333, \gamma =0.1,\sigma =1/4,\eta =0.5, q=0.0,\upzeta =0.0,{\omega }_{V}=0.0,{\omega }_{N}=0.0,m=0.1,{C}_{V}=0.5,\eta =0.5,A=0.0,a=0.5$ and $\theta =1/14$.

**Figure 16:**
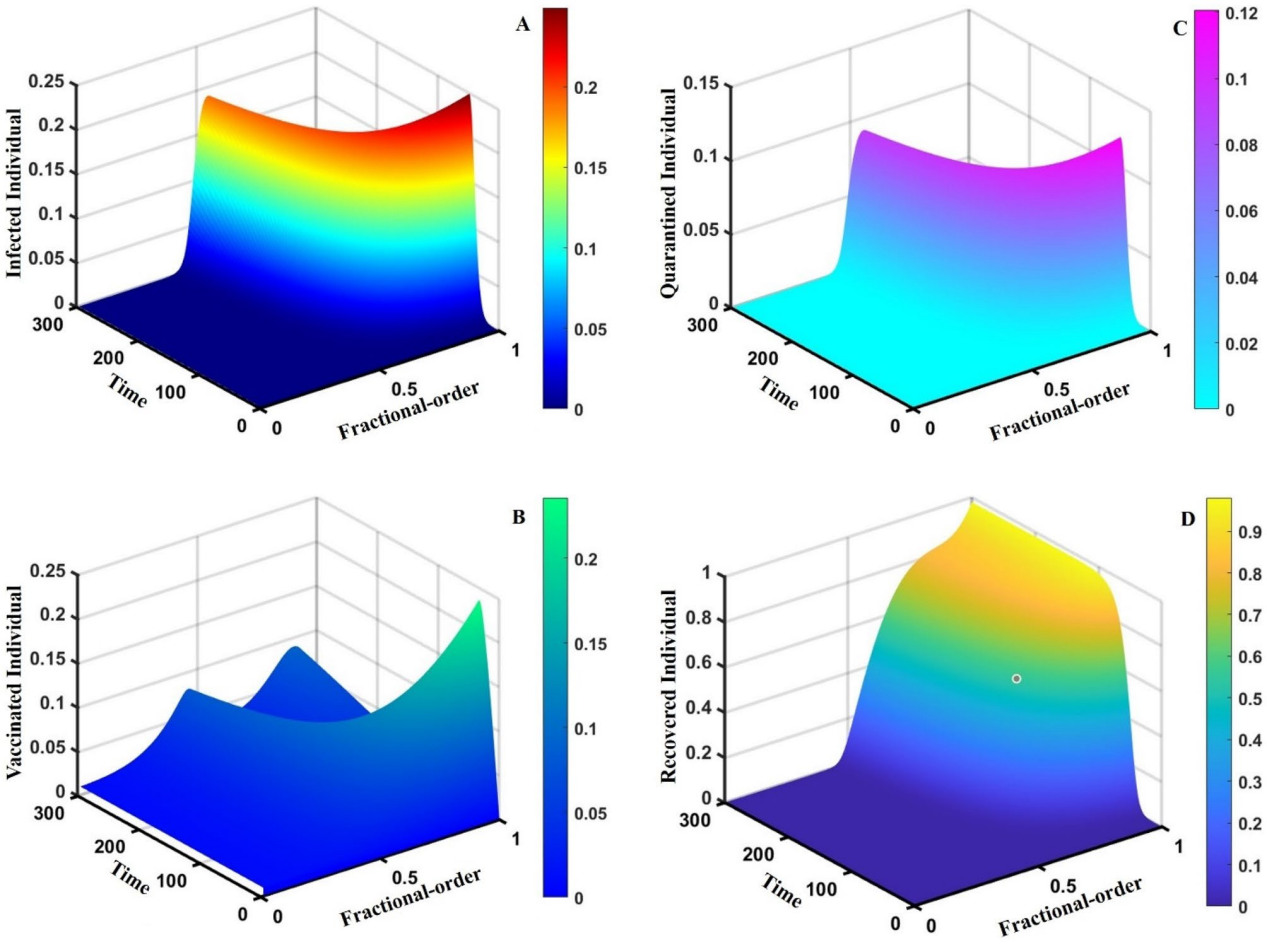
3D surface showing the effect of fractional order $(0\le \alpha \le 1)$ and time elapsed on the (**A**) infected, (**B**) vaccinated, (**C**) quarantined, and (**D**) recovered population, where parameters used are $\beta =0.8333, \gamma =0.1, \sigma =1/4,\eta =0.5, q=0.1,\upzeta =0.0,{\omega }_{V}=0.0,{\omega }_{N}=0.0,m=0.1,{C}_{V}=0.5,\eta =0.5,A=0.0,a=0.0$ and $\theta =1/14$.

**Figure 17:**
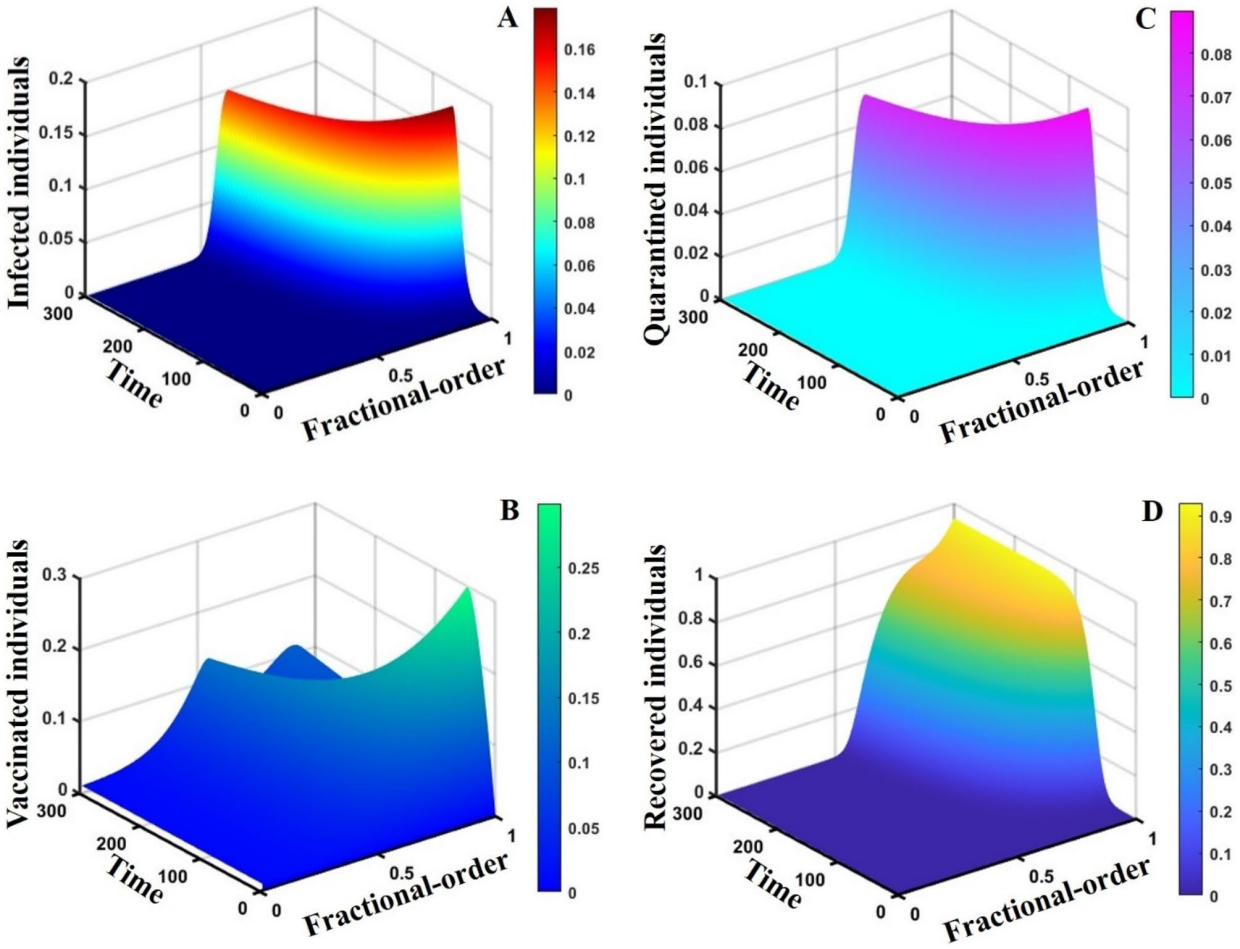
3D surface showing the effect of fractional order $(0\le \alpha \le 1)$ and time elapsed on the (**A**) infected, (**B**) vaccinated, (**C**) quarantined, and (**D**) recovered population, where parameters used are $\beta =0.8333, \gamma =0.1,\sigma =1/4,\eta =0.5, q=0.1,\upzeta =0.0,{\omega }_{V}=0.0,{\omega }_{N}=0.0,m=0.1,{C}_{V}=0.5,\eta =0.5,A=0.0,a=0.3,$ and $\theta =1/14$.

**Figure 18:**
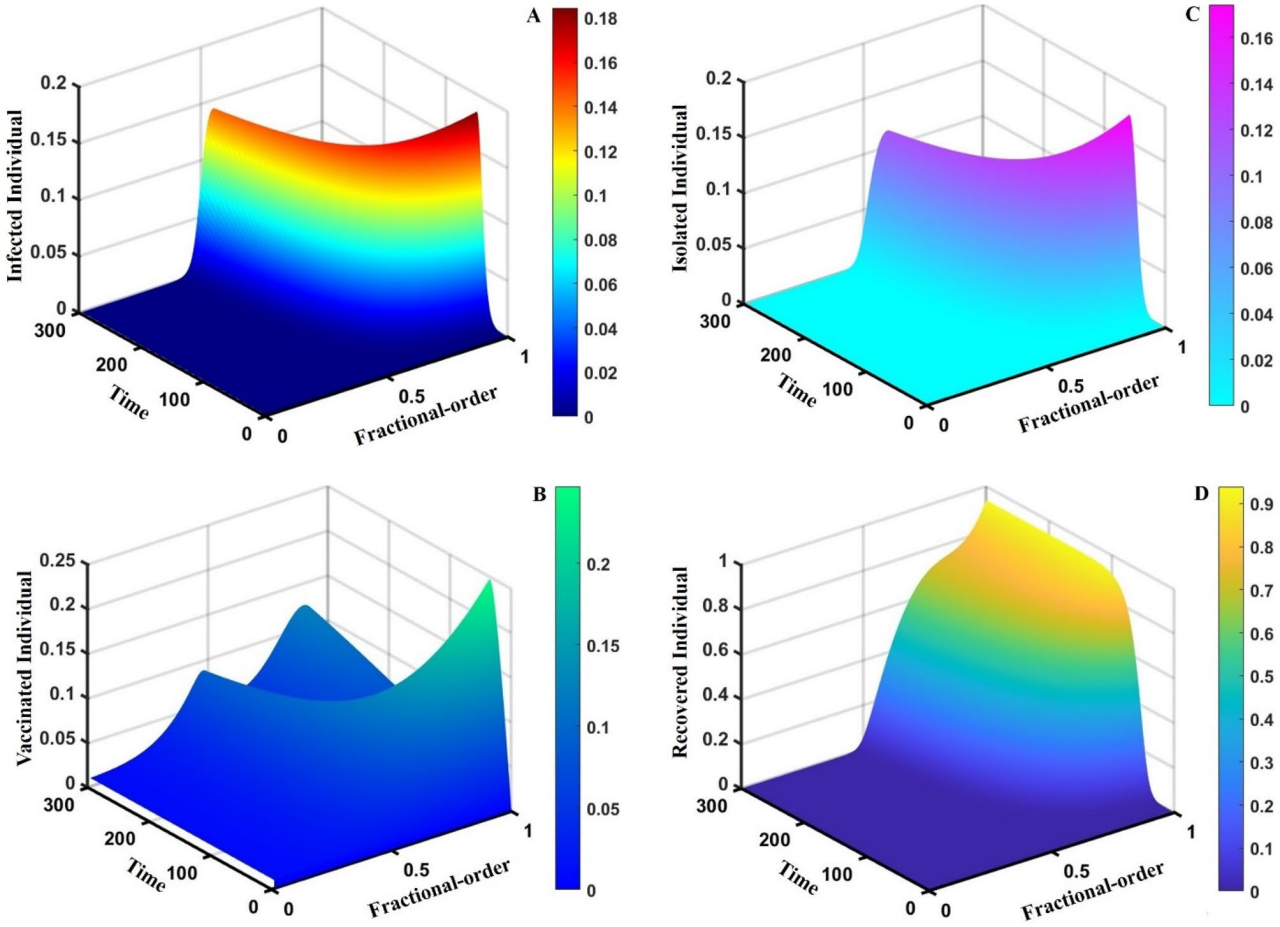
3D surface showing the effect of fractional order $(0\le \alpha \le 1)$ and time elapsed on the (**A**) infected, (**B**) vaccinated, (**C**) quarantined, and (**D**) recovered population, where parameters used are $\beta =0.8333, \gamma =0.1, \sigma =1/4,\eta =0.5, q=0.0,\upzeta =0.1,{\omega }_{V}=0.0,{\omega }_{N}=0.0,m=0.1,{C}_{V}=0.5,\eta =0.5,A=0.0,a=0.0,$ and $\theta =1/14$.

**Figure 19:**
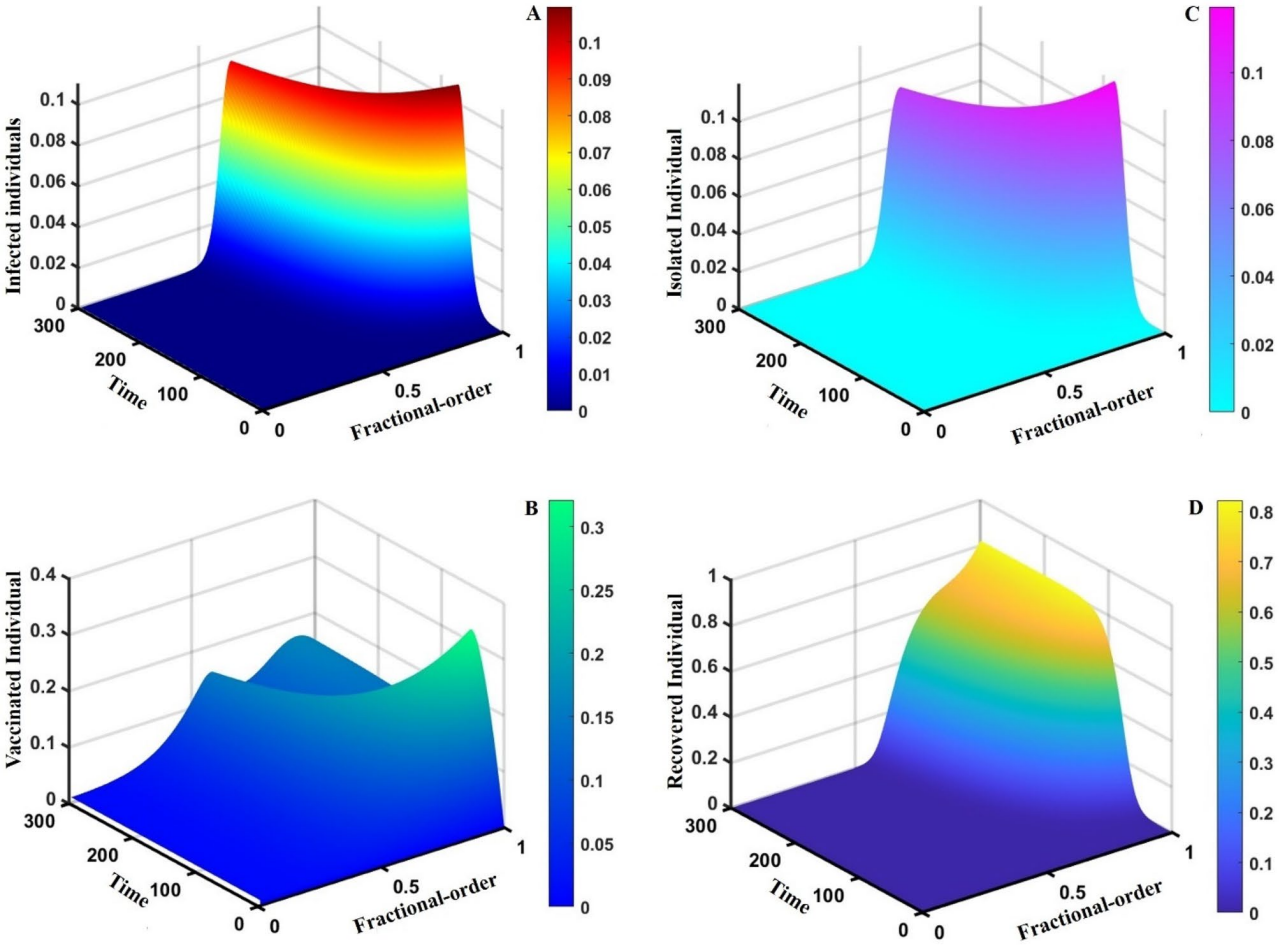
3D surface showing the effect of fractional order $(0\le \alpha \le 1)$ and time elapsed on the (**A**) infected, (**B**) vaccinated, (**C**) quarantined, and (**D**) recovered populations, where parameters used are $\beta =0.8333, \gamma =0.1,\sigma =1/4,\eta =0.5, q=0.0,\upzeta =0.1,{\omega }_{V}=0.0,{\omega }_{N}=0.0,m=0.1,{C}_{V}=0.5,\eta =0.5,A=0.0,a=0.3,$ and $\theta =1/14$.

**Figure 20:**
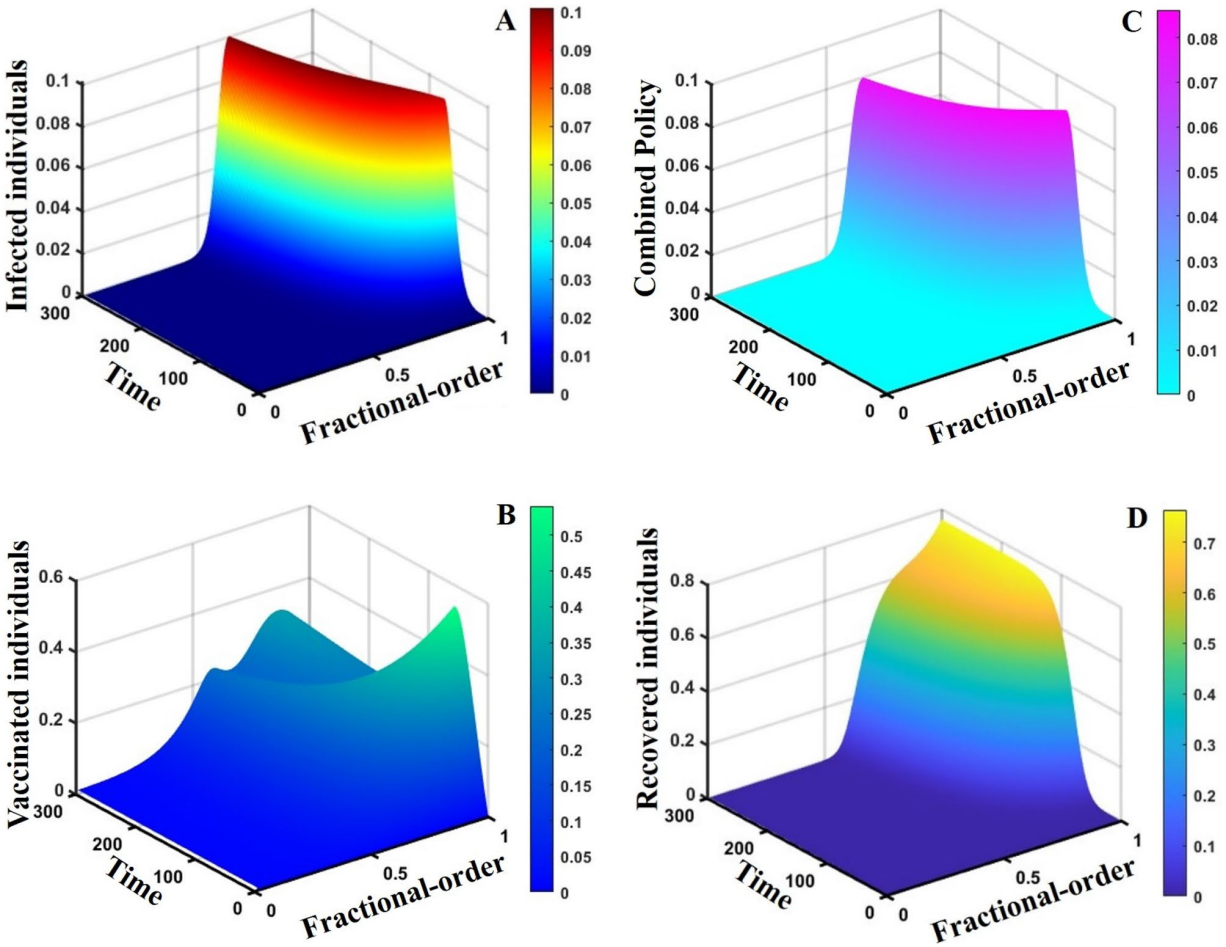
3D surface showing the effect of fractional order $(0\le \alpha \le 1)$ and time elapsed on the (**A**) infected, (**B**) vaccinated, (**C**) quarantined, and (**D**) recovered population, where parameters used are $\beta =0.8333, \gamma =0.1,\sigma =1/4,\eta =0.5, q=0.05,\upzeta =0.05,{\omega }_{V}=0.0,{\omega }_{N}=0.0,m=0.1,{C}_{V}=0.5,\eta =0.5,A=0.3,a=0.3,$ and $\theta =1/14$.

**Figure 21:**
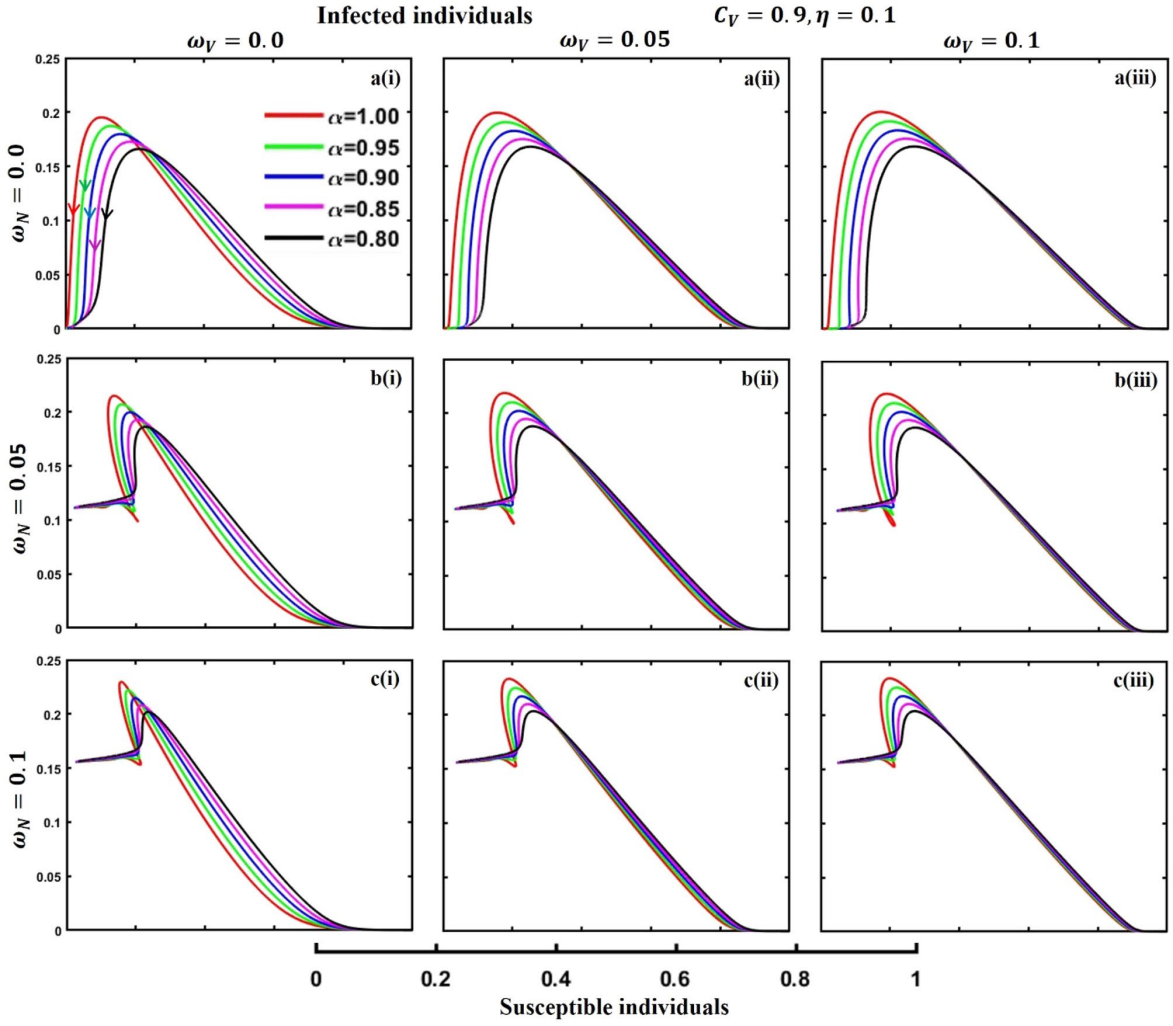
The phase-portrayed fractional order $(\alpha =\mathrm{0.8,0.85,0.9,0.95,1.0})$ trajectories of infected $(I(t))$ individuals concerning the waning rate of natural $\left({\omega }_{N}\right)$ and artificial $({\omega }_{V})$ immunity. Subpanels (a*), (b*), and (c*) show the naturally achieved immunity waning rate $\left({\omega }_{N}=0.0, 0.05, 0.1\right),$ whereas (*i), (*ii), (*iii) artificial immunity waning rate $({\omega }_{V}=0.0, 0.05, 0.1)$, respectively. The remaining parameters settings are $\beta =0.8333, \gamma =0.1,\sigma =1/4, q=0.1,\upzeta =0.1,m=0.1,{C}_{V}=0.9,\eta =0.1,A=0.0,a=0.0,$ and $\theta =1/14$.

**Figure 22:**
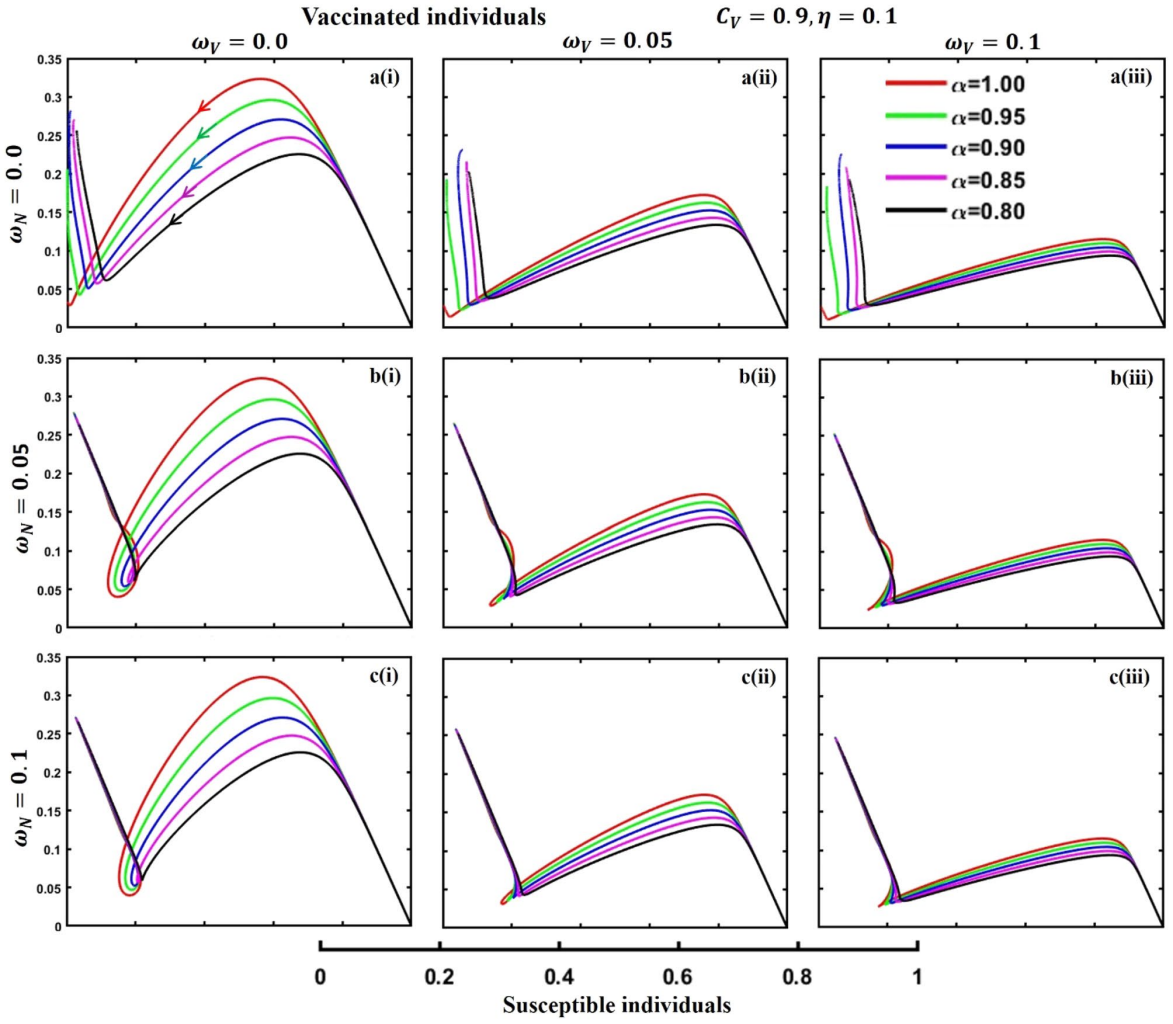
The phase-portrayed fractional order $(\alpha =\mathrm{0.8,0.85,0.9,0.95,1.0})$ trajectories of vaccinated $(V(t))$ individuals concerning the waning rate of natural $\left({\omega }_{N}\right)$ and artificial $({\omega }_{V})$ immunity. Subpanels (a*), (b*), and (c*) show the naturally achieved immunity waning rate $\left({\omega }_{N}=0.0, 0.05, 0.1\right),$ whereas (*i), (*ii), (*iii) artificial immunity waning rate $({\omega }_{V}=0.0, 0.05, 0.1)$, respectively. The remaining parameters settings are $\beta =0.8333, \gamma =0.1, \sigma =1/4, q=0.1,\upzeta =0.1,m=0.1,{C}_{V}=0.9,\eta =0.1,A=0.0,a=0.0,$ and $\theta =1/14$.

**Figure 23:**
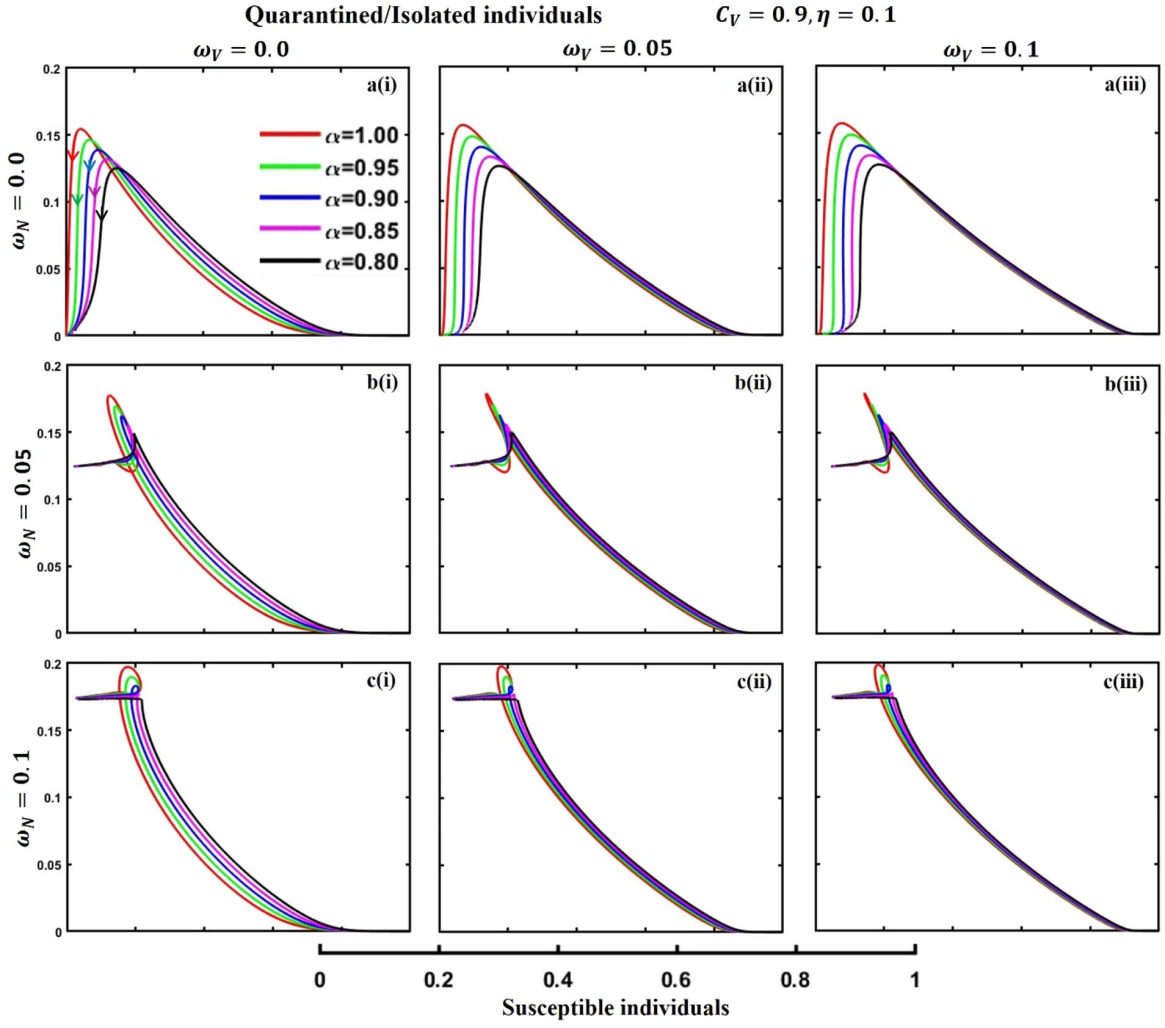
The phase-portrayed fractional order $(\alpha =\mathrm{0.8,0.85,0.9,0.95,1.0})$ trajectories of quarantined and isolated $(J(t))$ individuals concerning the waning rate of natural $\left({\omega }_{N}\right)$ and artificial $({\omega }_{V})$ immunity. Subpanels (a*), (b*), and (c*) show the naturally achieved immunity waning rate $\left({\omega }_{N}=0.0, 0.05, 0.1\right),$ whereas (*i), (*ii), (*iii) artificial immunity waning rate $({\omega }_{V}=0.0, 0.05, 0.1)$, respectively. The remaining parameters settings are $\beta =0.8333, \gamma =0.1, \sigma =1/4, q=0.1,\upzeta =0.1,m=0.1,{C}_{V}=0.9,\eta =0.1,A=0.0,a=0.0,$ and $\theta =1/14$.

**Figure 24:**
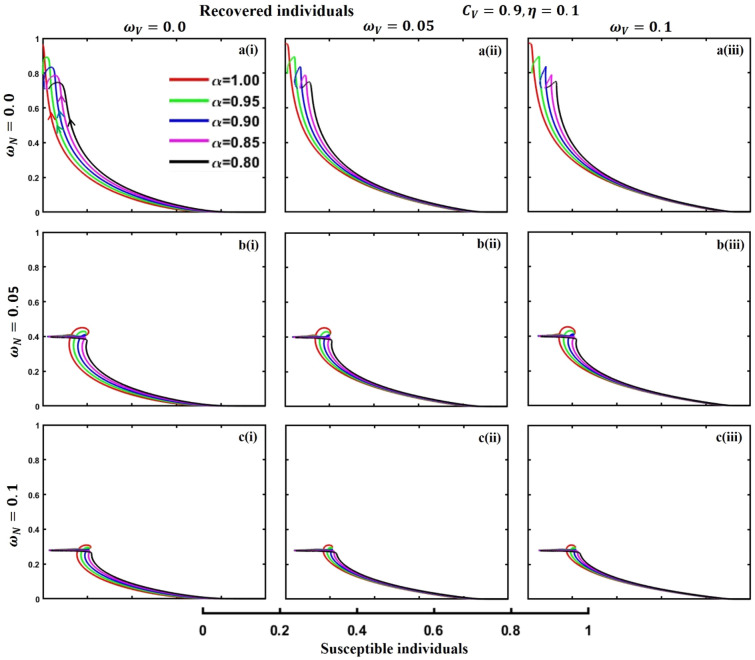
The phase-portrayed fractional order $(\alpha =\mathrm{0.8,0.85,0.9,0.95,1.0})$ trajectories of recovered $(R(t))$ individuals concerning the waning rate of natural $\left({\omega }_{N}\right)$ and artificial $({\omega }_{V})$ immunity. Subpanels (a*), (b*), and (c*) show the naturally achieved immunity waning rate $\left({\omega }_{N}=0.0, 0.05, 0.1\right),$ whereas (*i), (*ii), (*iii) artificial immunity waning rate $({\omega }_{V}=0.0, 0.05, 0.1)$, respectively. The remaining parameters settings are $\beta =0.8333, \gamma =0.1, \sigma =1/4, q=0.1,\upzeta =0.1,m=0.1,{C}_{V}=0.9,\eta =0.1,A=0.0,a=0.0,$ and $\theta =1/14$.

**Figure 25:**
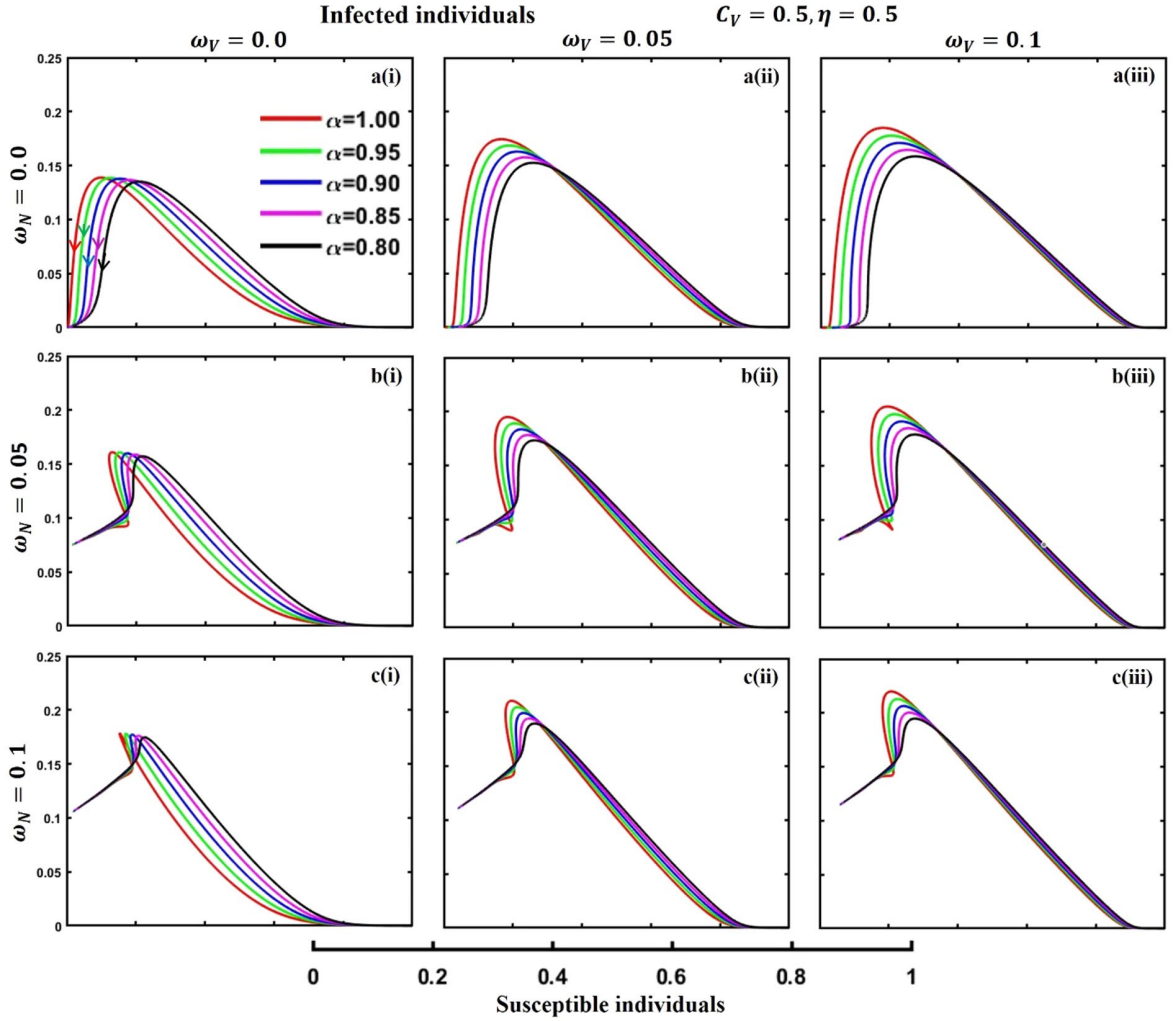
The phase-portrayed fractional order $(\alpha =\mathrm{0.8,0.85,0.9,0.95,1.0})$ trajectories of infected $(I(t))$ individuals concerning the waning rate of natural $\left({\omega }_{N}\right)$ and artificial $({\omega }_{V})$ immunity. Subpanels (a*), (b*), and (c*) show the naturally achieved immunity waning rate $\left({\omega }_{N}=0.0, 0.05, 0.1\right),$ whereas (*i), (*ii), (*iii) artificial immunity waning rate $({\omega }_{V}=0.0, 0.05, 0.1)$, respectively. The remaining parameters settings are $\beta =0.8333, \gamma =0.1,\sigma =1/4, q=0.1,\upzeta =0.1,m=0.1,{C}_{V}=0.5,\eta =0.5,A=0.0,a=0.0,$ and $\theta =1/14$.

**Figure 26:**
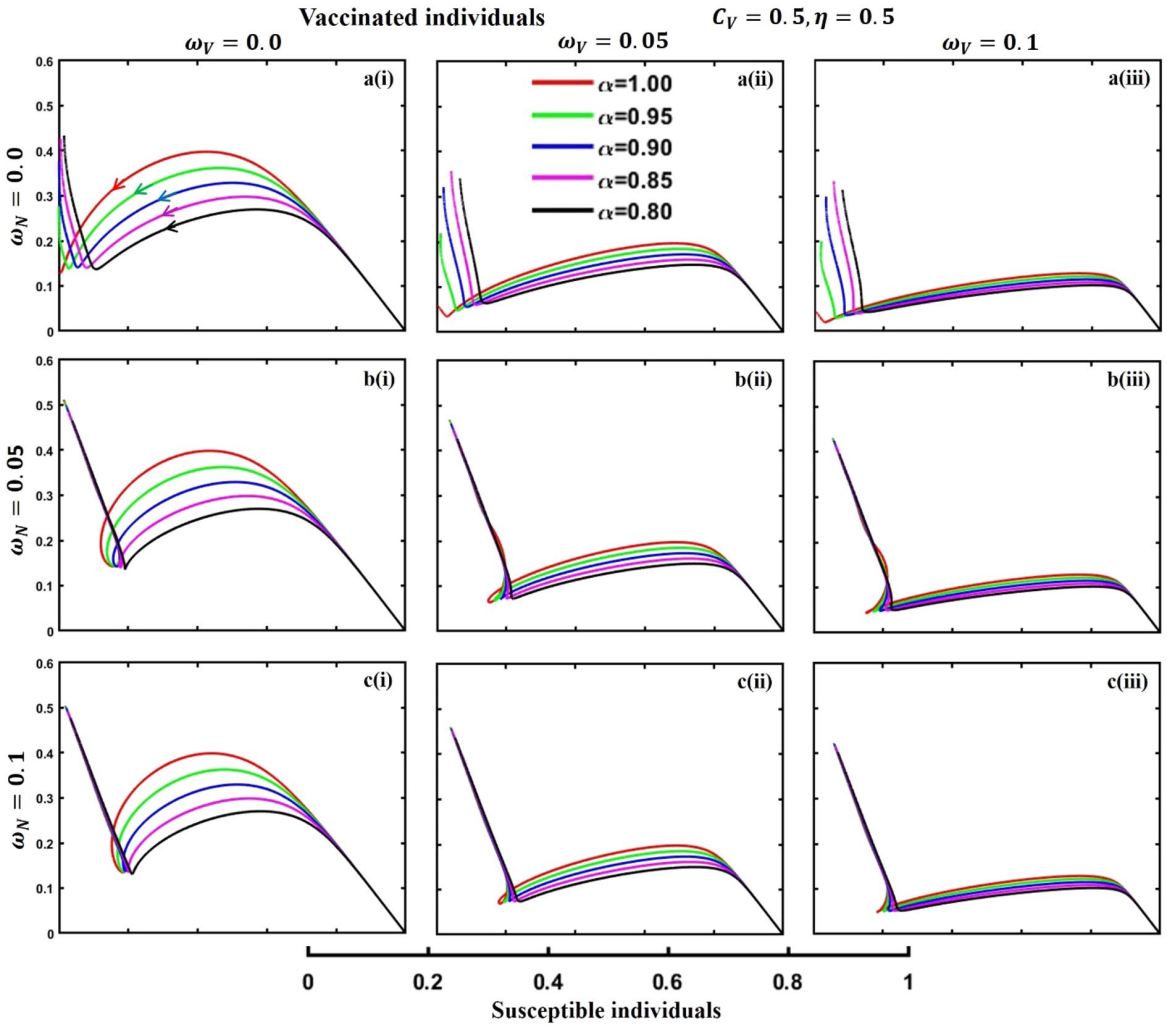
The phase-portrayed fractional order $(\alpha =\mathrm{0.8,0.85,0.9,0.95,1.0})$ trajectories of vaccinated $(V(t))$ individuals concerning the waning rate of natural $\left({\omega }_{N}\right)$ and artificial $({\omega }_{V})$ immunity. Subpanels (a*), (b*), and (c*) show the naturally achieved immunity waning rate $\left({\omega }_{N}=0.0, 0.05, 0.1\right),$ whereas (*i), (*ii), (*iii) artificial immunity waning rate $({\omega }_{V}=0.0, 0.05, 0.1)$, respectively. The remaining parameters settings are $\beta =0.8333, \gamma =0.1, \sigma =1/4, q=0.1,\upzeta =0.1,m=0.1,{C}_{V}=0.5,\eta =0.5,A=0.0,a=0.0,$ and $\theta =1/14$.

**Figure 27:**
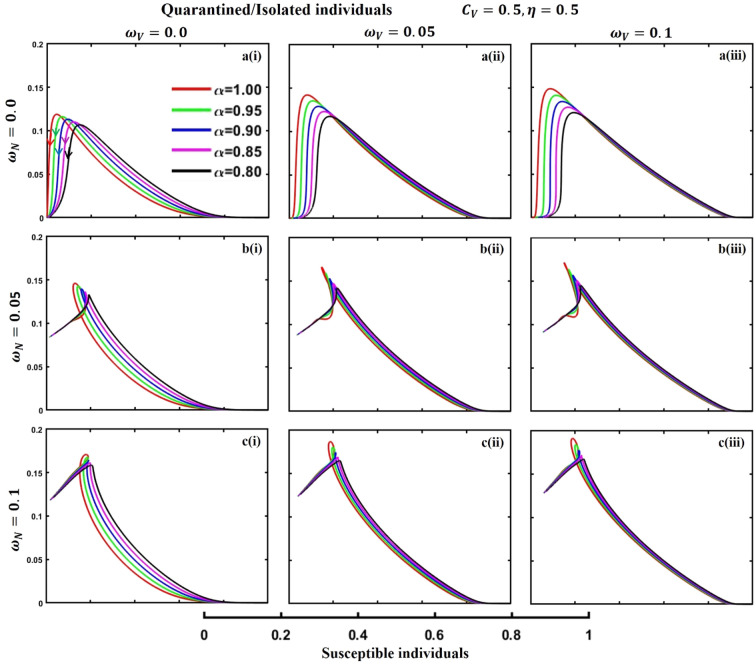
The phase-portrayed fractional order $(\alpha =\mathrm{0.8,0.85,0.9},\mathrm{0.95,1.0})$ trajectories of quarantined and Isolated $(J(t))$ individuals concerning the waning rate of natural $\left({\omega }_{N}\right)$ and artificial $({\omega }_{V})$ immunity. Subpanels (a*), (b*), and (c*) show the naturally achieved immunity waning rate $\left({\omega }_{N}=0.0, 0.05, 0.1\right),$ whereas (*i), (*ii), (*iii) artificial immunity waning rate $({\omega }_{V}=0.0, 0.05, 0.1)$, respectively. The remaining parameters settings are $\beta =0.8333, \gamma =0.1, \sigma =1/4, q=0.1,\upzeta =0.1,m=0.1,{C}_{V}=0.5,\eta =0.5,A=0.0,a=0.0,$ and $\theta =1/14$.

**Figure 28:**
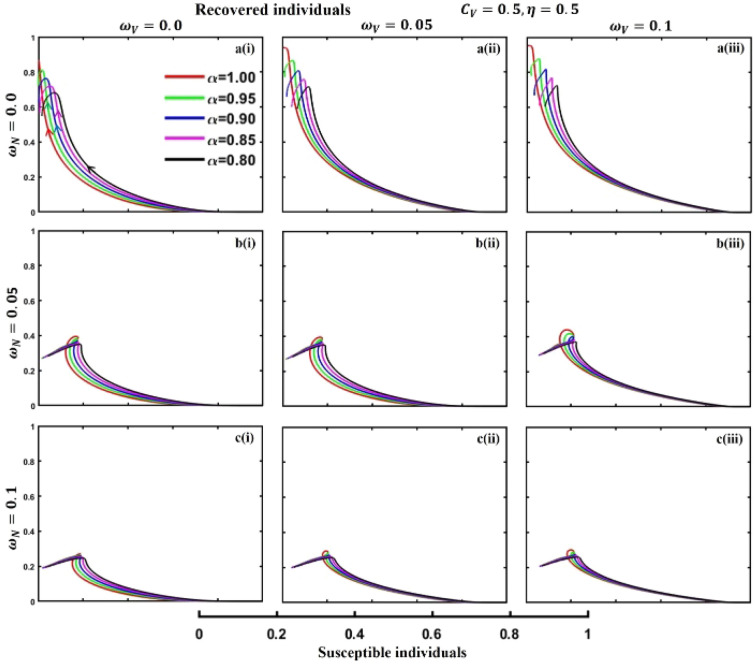
The phase-portrayed fractional order $(\alpha =\mathrm{0.8,0.85,0.9,0.95,1.0})$ trajectories of recovered $(R(t))$ individuals concerning the waning rate of natural $\left({\omega }_{N}\right)$ and artificial $({\omega }_{V})$ immunity. Subpanels (a*), (b*), and (c*) show the naturally achieved immunity waning rate $\left({\omega }_{N}=0.0, 0.05, 0.1\right),$ whereas (*i), (*ii), (*iii) artificial immunity waning rate $({\omega }_{V}=0.0, 0.05, 0.1)$, respectively. The remaining parameters settings are $\beta =0.8333, \gamma =0.1, \sigma =1/4, q=0.1,\upzeta =0.1,m=0.1,{C}_{V}=0.5,\eta =0.5,A=0.0,a=0.0,$ and $\theta =1/14$.

**Figure 29:**
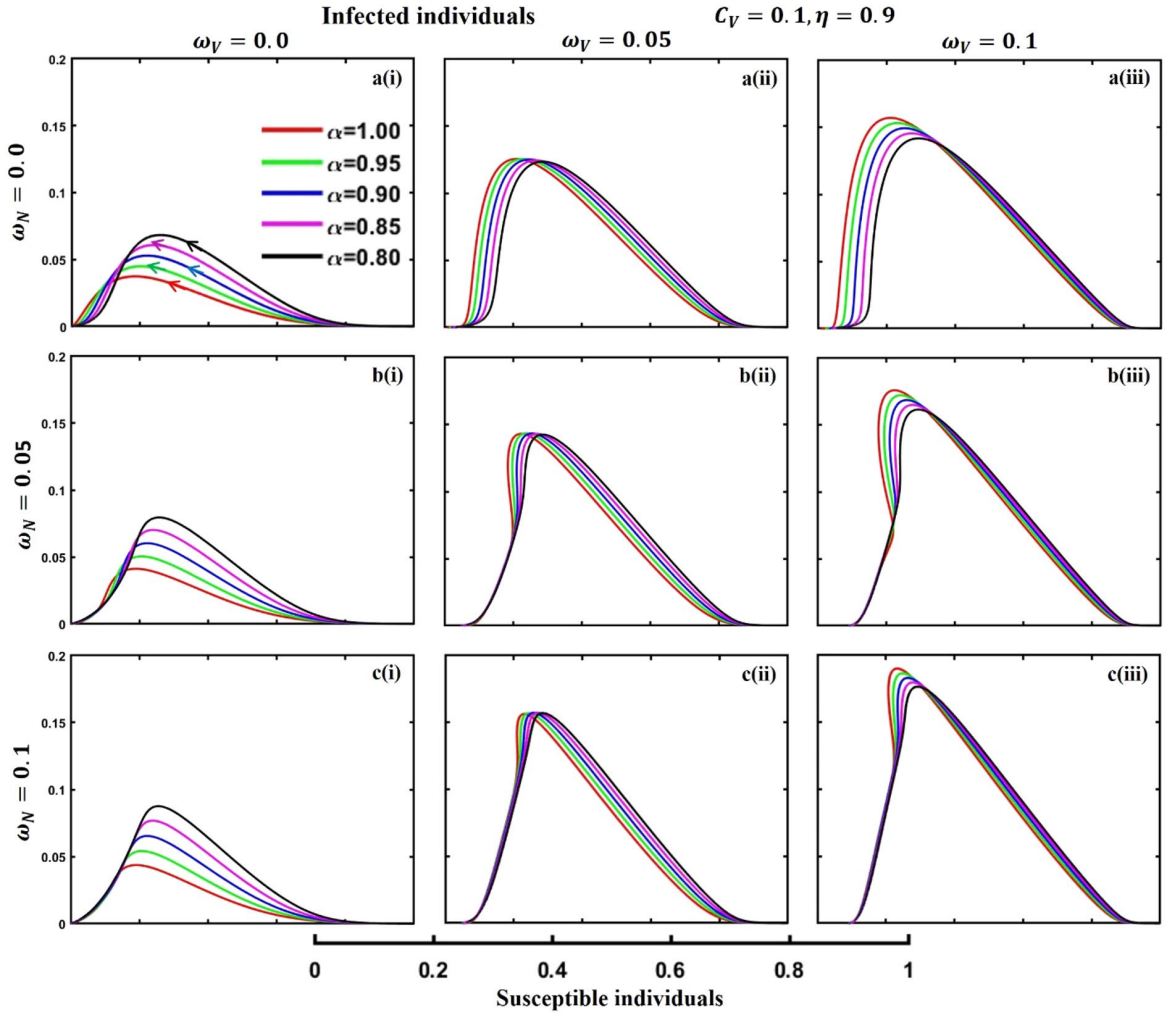
The phase-portrayed fractional order $(\alpha =\mathrm{0.8,0.85,0.9,0.95,1.0})$ trajectories of infected $(I(t))$ individuals concerning the waning rate of natural $\left({\omega }_{N}\right)$ and artificial $({\omega }_{V})$ immunity. Subpanels (a*), (b*), and (c*) show the naturally achieved immunity waning rate $\left({\omega }_{N}=0.0, 0.05, 0.1\right),$ whereas (*i), (*ii), (*iii) artificial immunity waning rate $({\omega }_{V}=0.0, 0.05, 0.1)$, respectively. The remaining parameters settings are $\beta =0.8333, \gamma =0.1, \sigma =1/4, q=0.1,\upzeta =0.1,m=0.1,{C}_{V}=0.1,\eta =0.9,A=0.0,a=0.0,$ and $\theta =1/14$.

**Figure 30:**
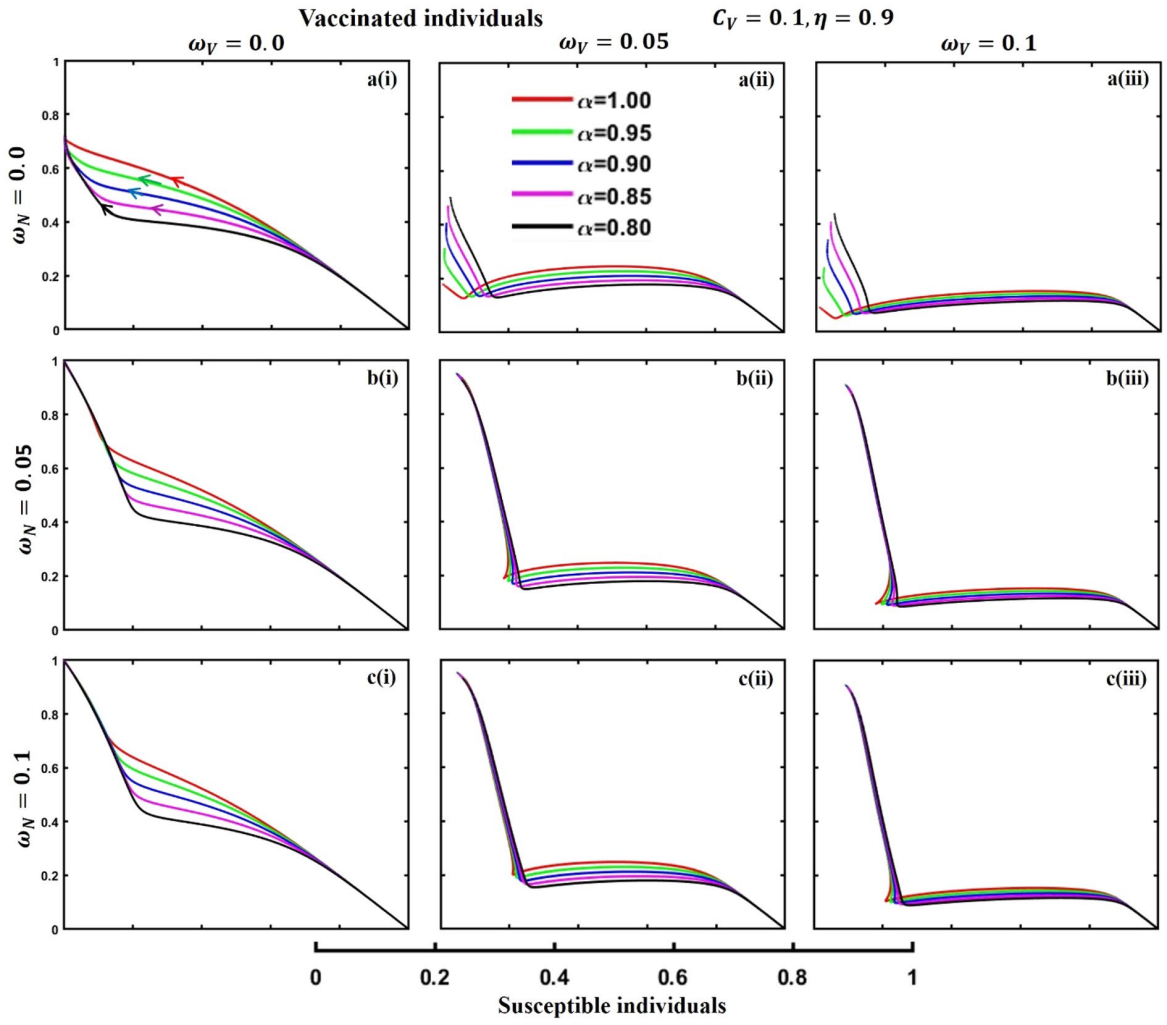
The phase-portrayed fractional order $(\alpha =\mathrm{0.8,0.85,0.9,0.95,1.0})$ trajectories of vaaccinated $(V(t))$ individuals concerning the waning rate of natural $\left({\omega }_{N}\right)$ and artificial $({\omega }_{V})$ immunity. Subpanels (a*), (b*), and (c*) show the naturally achieved immunity waning rate $\left({\omega }_{N}=0.0, 0.05, 0.1\right),$ whereas (*i), (*ii), (*iii) artificial immunity waning rate $({\omega }_{V}=0.0, 0.05, 0.1)$, respectively. The remaining parameters settings are $\beta =0.8333, \gamma =0.1, \sigma =1/4, q=0.1,\upzeta =0.1,m=0.1,{C}_{V}=0.1,\eta =0.9,A=0.0,a=0.0,$ and $\theta =1/14$.

**Figure 31:**
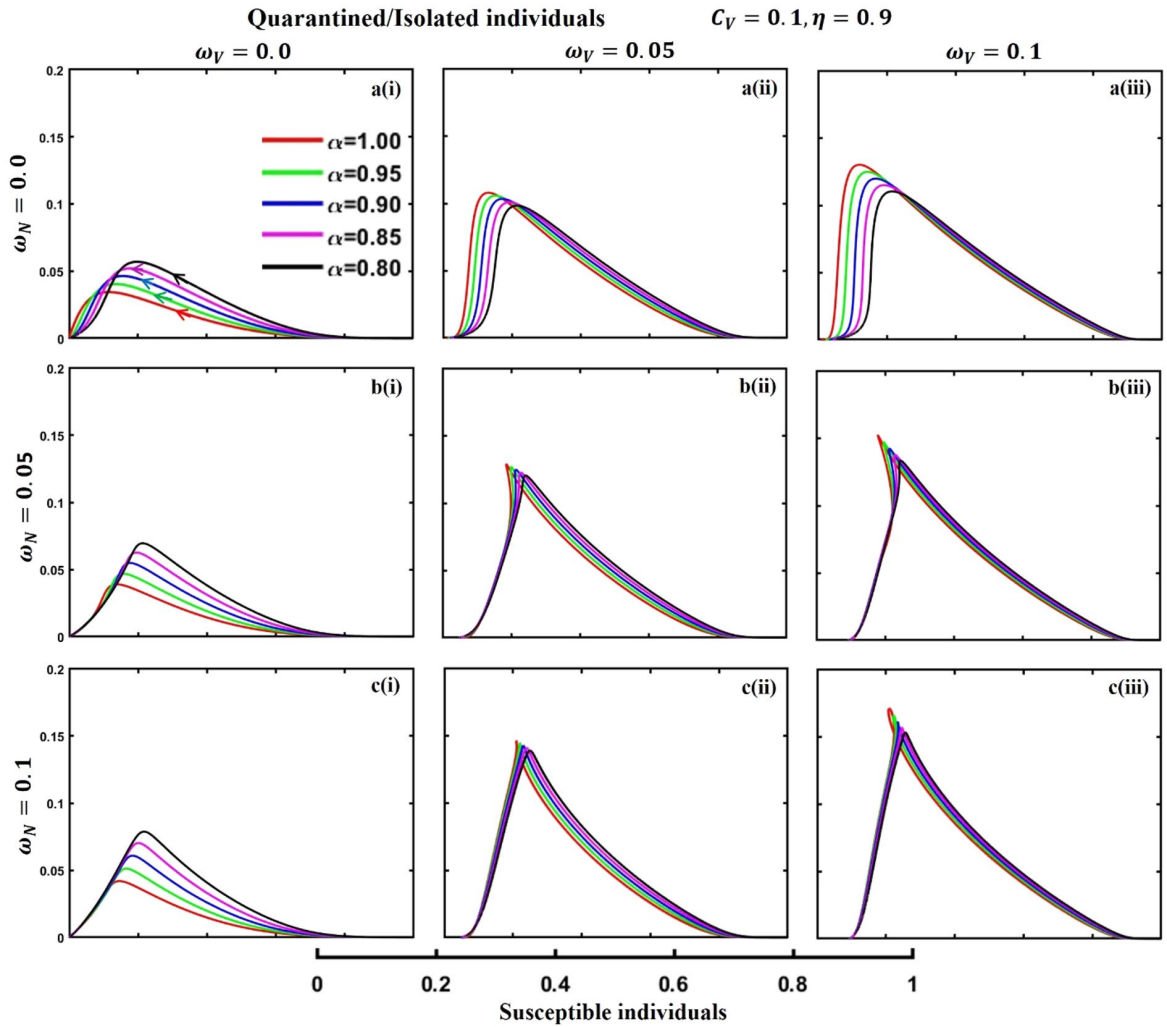
The phase-portrayed fractional order $(\alpha =\mathrm{0.8,0.85,0.9,0.95,1.0})$ trajectories of quarantined and isolated $(J(t))$ individuals concerning the waning rate of natural $\left({\omega }_{N}\right)$ and artificial $({\omega }_{V})$ immunity. Subpanels (a*), (b*), and (c*) show the naturally achieved immunity waning rate $\left({\omega }_{N}=0.0, 0.05, 0.1\right),$ whereas (*i), (*ii), (*iii) artificial immunity waning rate $({\omega }_{V}=0.0, 0.05, 0.1)$, respectively. The remaining parameters settings are $\beta =0.8333, \gamma =0.1, \sigma =1/4, q=0.1,\upzeta =0.1,m=0.1,{C}_{V}=0.1,\eta =0.9,A=0.0,a=0.0,$ and $\theta =1/14$.

**Figure 32:**
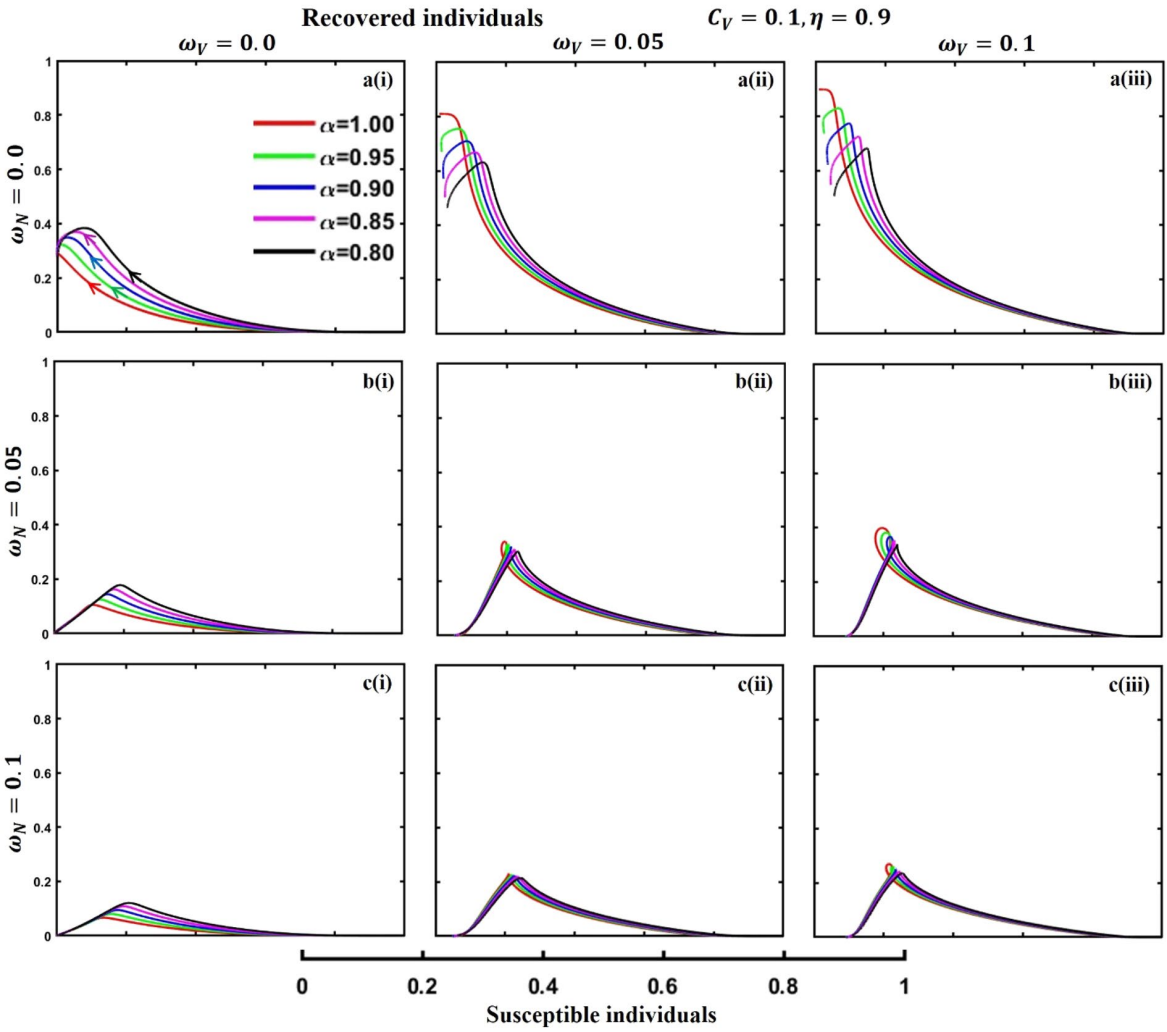
The phase-portrayed fractional order $(\alpha =\mathrm{0.8,0.85,0.9,0.95,1.0})$ trajectories of recovered $(R(t))$ individuals concerning the waning rate of natural $\left({\omega }_{N}\right)$ and artificial $({\omega }_{V})$ immunity. Subpanels (a*), (b*), and (c*) show the naturally achieved immunity waning rate $\left({\omega }_{N}=0.0, 0.05, 0.1\right),$ whereas (*i), (*ii), (*iii) artificial immunity waning rate $({\omega }_{V}=0.0, 0.05, 0.1)$, respectively. The remaining parameters settings are $\beta =0.8333, \gamma =0.1,\sigma =1/4, q=0.1,\upzeta =0.1,m=0.1,{C}_{V}=0.1,\eta =0.9,A=0.0,a=0.0,$ and $\theta =1/14$.

This section elaborates on the proposed epidemic model’s disease transmission dynamics for different parameter settings with fractional order $\alpha =\mathrm{0.8,0.85,0.9,0.95,1.0}$. The Atangana–Baleanu–Caputo (ABC) fractional-order derivative algorithm^32^ is used to conduct the numerical simulation of the proposed awareness-based quarantine/isolation and vaccination model. Firstly, our attention was on the graphical representation of the numerical simulation results for the time-evolving curve (Figs. 2, 3, 4, 5, 6, 7, 8, 9, 10, 11, 12 and 13) of the suggested fractional-order model’s endemic stability with and without the evolutionary game theoretical strategy. Then, we epitomize 3D surface diagrams (Figs. 14, 15, 16, 17, 18, 19 and 20) to demonstrate diseases’ overall dynamics concerning changing fractional order $0\le \alpha \le 1$. Finally, we first represent a phase-portrayed trajectory (Figs. 21, 22, 23, 24, 25, 26, 27, 28, 29, 30, 31 and 32) graph for artificial and natural immunity waning through a fractional derivative approach concerning three different settings of vaccination costs $({C}_{V})$ and vaccine efficacy $(\eta )$. The time mentioned above series with different fractional order plays a crucial role in understanding, monitoring, and controlling diseases. It provides significant information on the dynamics of the disease, helps identify epidemics at an early stage, delays effects, and allows for evaluating the effectiveness of interventions.

When any disease emerges in any part of the world, awareness about the disease is crucial to being safe from the disease. Also, it helps individuals to alleviate its transmission in other areas. Figure 2, subpanels (a*-), (b*-), and (c*-) represent the effect of personal awareness parameters during an epidemic or pandemic time on infected, vaccinated, and recovered individuals concerning different fractional-order $(\alpha =\mathrm{0.8,0.85,0.9,0.95,1.0})$ values. In subpanel (a*-), it is crystal clear that the infected individual’s curve demonstrates a decreasing trend with a slowing of the epidemic peak due to the increasing rate of the personal awareness parameter value. In a real-world situation, in any epidemic or pandemic, if people are more aware of the diseases or have enough information about the disease, then disease transmission cannot spread quickly, which shows a delayed scenario. It takes much time to reach its highest peak. As a result, policymakers have enough scope to invent proper vaccines or medicine to combat the disease. Again, when people are more aware of the disease, they participate in vaccination programs, and such scenarios are demonstrated in subpanel (b*-). Aware humans know non-pharmaceutical interventions are not a permanent solution compared to pharmaceutical interventions, such as vaccination. As a result, when people are more aware, they participate more in vaccination programs. On the other hand, when people are more aware, disease fails to spread to all parts of society and cannot show its hazardous magnitudes, and the recovered curve must show a decreasing tendency according to subpanel (c*-), an expected outcome.

Efficient quarantine policies and comprehensive vaccination strategies are crucial tools in fighting the transmission of contagious diseases. Quarantine techniques imposing limitations on the movement of persons exposed to or infected with a contagious virus effectively contain outbreaks and prevent the pathogen’s spread within populations. Furthermore, vaccination is crucial in establishing herd immunity, diminishing the population’s vulnerability to the disease, and ultimately curbing its transmission. Public health authorities can effectively reduce the impact of infectious diseases by implementing stringent vaccination programs in conjunction with focused quarantine measures. This approach protects the health of individuals and the general public and works towards eliminating diseases and preventing epidemics or pandemics. Figure 3, subpanels (a*-), (b*-), and (c*-) signify the outcome of quarantine rate $q=0.1$ and $q=0.2$ during an epidemic or pandemic on infected, vaccinated, and recovered individuals concerning different fractional-order $(\alpha =\mathrm{0.8,0.85,0.9,0.95,1.0})$ values. A higher quarantine rate decreases the infected (subpanel (a*-)) and recovered (subpanel (c*-)) individuals scenarios and increases the vaccinated (subpanel (b*-)) people’s outcomes. In the natural world, when individuals effectively maintain a quarantine policy, the possibility of disease spreading reduces at a specific rate, reducing the final epidemic size (FES) and increasing vaccination coverage (VC).

Effective isolation policies are essential when combined with vaccination measures to fight the transmission of contagious diseases. Isolation procedures include the segregation of persons who are affected from the broader community in order to hinder the spread of the disease. This can include isolating persons who exhibit symptoms or have been confirmed to be infected with the disease and employing more extensive measures to maintain physical distance in society. The efficiency of managing infectious diseases is greatly improved when vaccination tactics are used to develop immunity in the community. Nevertheless, the implementation of isolation rules remains essential, particularly during periods of epidemics or in situations when the distribution of vaccines is not yet extensive. Thus, in Fig. 4, subpanels (a*-), (b*-), and (c*-) reveal the consequence of isolation rate $\zeta =0.1$ and $\zeta =0.2$ during an epidemic or pandemic on infected, vaccinated, and recovered individuals concerning different fractional-order $(\alpha =\mathrm{0.8,0.85,0.9,0.95,1.0})$ values. A higher rate of perfect isolation policy alleviates the disease outbreak (subpanel (a*-)). Also, it allows individuals to participate in vaccination campaigns. As a result, it prevents the pathogen’s spread within populations and shows a lower FES (subpanel (c*-)) and higher VC (subpanel (b*-)). Therefore, with the quick and efficient isolation of affected persons, the transmission of infectious diseases can be limited, serving as a vital defensive mechanism in addition to vaccination initiatives. In addition, if we compare the results of quarantine and isolation policies, more precisely, the outcomes demonstrated in Figs. 3 and 4, it is evident that the isolation policy is better than the quarantine policy and the vaccination strategy.

Policies of isolation and quarantine, predominantly in aggregation with effective vaccination strategies $(\eta =\mathrm{0.5,0.9})$, are essential for the fight against the spread of infectious diseases as demonstrated in Fig. 5, subpanels (a*-), (b*-), and (c*-) with different fractional-order $(\alpha =\mathrm{0.8,0.85,0.9,0.95,1.0})$ values. Separating and limiting the mobility of people exposed to a contagious disease but may not yet exhibit symptoms is known as quarantine while isolating afflicted persons to stop further transmission is known as isolation. These steps are essential for stopping epidemics and safeguarding vulnerable groups of people. Figures 3 and 4 separately elucidate the outcomes of quarantine and isolation policy and establish that isolation policy is a better tool to combat epidemics or pandemics. So, combining both strategies must be an excellent tactic to battle any epidemics or pandemics, and Fig. 5 exhibits such types of outcomes. When the value of vaccine efficacy is $\eta =0.9$, Fig. 5a(ii) demonstrates one of the fundamental characteristics of fractional-order derivative, where deterministic processes fail to describe such scenarios that the lower order fractional-order derivative shows the highest pinnacle point of the epidemic. However, extensive immunization efforts with highly effective vaccines are crucial to gaining herd immunity. Vaccination inhibits the spread of the disease throughout communities and lowers the risk that a person may develop it. Public health authorities can substantially reduce the burden of infectious diseases and eventually prevent future outbreaks by prioritizing vaccination efforts, especially targeting high-risk populations and ensuring equal access. Furthermore, public education and awareness efforts are necessary to encourage adherence to isolation, vaccination, and quarantine guidelines and create a shared responsibility for disease prevention and control.

Individual consciousness and stringent quarantine and isolation measures are fundamental in the fight against contagious diseases. Increased awareness of hygiene measures, symptom identification, and adherence to public health standards significantly curtail disease transmission chains. Simultaneously, strict quarantine and isolation procedures efficiently restrict transmission spread, avoiding large-scale epidemics. Nevertheless, the effectiveness of these strategies can be greatly enhanced with comprehensive vaccination methods. By guaranteeing universal access to vaccinations and raising vaccination rates via educational and outreach initiatives, communities can create herd immunity, strengthening their resistance to infectious pathogens. A robust defense is created to combat the spread of infectious diseases through the symbiotic integration of individual vigilance and public health activities. Figure 6, subpanels (a*-), (b*-), and (c*-) reveal the impact fractional-order $(\alpha =\mathrm{0.8,0.85,0.9,0.95,1.0})$ to demonstrate the significance of personal awareness $a=0.5$, quarantine rate ${q}_{1}=0.1$ and isolation rate ${q}_{2}=0.2$ with vaccine efficacy $\eta =0.5$ and $\eta =0.8$ during an epidemic or pandemic on infected, vaccinated, and recovered individuals. It is evident that Fig. 6a(ii) demonstrates that disease almost dies out when the vaccine’s efficacy is $\eta =0.8$ and shows the same characteristics as Fig. 5a(ii). Also, the number of infected people (Fig. 6a(ii)) is so minimal that the FES is small in size (Fig. 6c(ii)).

The active involvement of government forces, primarily the active participation of individuals in vaccination programs, is crucial in controlling infectious diseases. Government agencies promote broad access to vaccinations and strengthen community immunity by mobilizing resources, coordinating logistics, and executing targeted initiatives. These entities use public health campaigns, educational activities, and outreach endeavors to encourage the adoption and adherence to vaccines, reducing the transmission of infectious diseases and safeguarding populations at risk. Government bodies use their power and experience to promote cooperation among healthcare practitioners, researchers, and policymakers, facilitating a cohesive disease prevention approach. Ultimately, their active participation enhances the public health system and protects communities from the risk of contagious diseases. Achieving the outcome as mentioned above, we demonstrated Figs. 7, 8 and 9, subpanels (a*-), (b*-), and (c*-) for different values of government force parameter $A=\mathrm{0.1,0.5}$ and 0.9 in the sense of vaccine cost and efficacy ${(C}_{V},\eta )=\left(\mathrm{0.9,0.1}\right),(\mathrm{0.5,0.5})$ and $(\mathrm{0.1,0.9})$ to signify the impact of said parameters on infected, vaccinated, and recovered individuals during an epidemic or pandemic through fractional order $(\alpha =\mathrm{0.8,0.85,0.9,0.95,1.0})$ derivative. When vaccination costs are high, but efficacy is low due to government forces, people participate in vaccination programs (Fig. 7, subpanels (b*-)) that do not significantly impact disease transmission (Fig. 7, subpanels (a*-)). A better situation of disease will occur for ${(C}_{V},\eta )=\left(\mathrm{0,5},0.5\right)$ and favorable for ${(C}_{V},\eta )=\left(\mathrm{0.1,0.9}\right)$. In the third setting of vaccination cost and efficacy, we observed the opposite scenario for infected individuals (Fig. 9, subpanels (a*-)) compared to the first two (Figs. 7, 8, subpanels (a*-)), that lower-order fractional derivative (black line, $\alpha =0.8$) shows the peak of the epidemic. It is well established that any suitable vaccination program significantly sets a barrier to the disease transmission mechanism; as a result, the disease will slow down, and we can observe the epidemic peak for lower order fractional value. Finally, at the mass level, it is pretty challenging to maintain different non-pharmaceutical interventions, so in the absence of individual awareness, quarantine, or isolation policy, policymakers always try to adopt permanent solutions to control the spread of any epidemic or pandemic. In that situation, a highly effective and less costly vaccination program is crucial to curbing the spread of infectious diseases and preventing diseases from spreading in society.

Figures 10, 11, 12 and 13 displayed the outcomes of personal awareness, quarantine, and quarantine and isolation policy in the context of the EGT mechanism of vaccination cost and efficacy ${(C}_{V},\eta )=\left(\mathrm{0.9,0.1}\right) {\text{and}} (\mathrm{0.1,0.9})$ and fractional order $(\alpha =\mathrm{0.8,0.85,0.9,0.95,1.0})$ derivative approach. If we observe the infected curve of Figs. 10, 11, 12 and 13, it is evident that personal awareness (Fig. 10, subpanels (a*-)) plays a significant role in reducing disease transmission compared to quarantine (Fig. 11, subpanels (a*-)) and isolation (Fig. 12, subpanels (a*-)) policy with vaccination and it delayed the epidemic peak, which represented by a black line $(\alpha =0.8)$. Realistically, when people are unaware of any disease, they must pay awareness and vaccination costs. Therefore, awareness is a must to protect from any disease, as pharmaceutical and non-pharmaceutical interventions are not a permanent solution. Again, if we compare the result of Figs. 10, 11 and 12, subpanels (a*-) with (Fig. 13, subpanels (a*-)), we see that the combined quarantine and isolation policy is a better indicator to curtail the disease from society with EGT setting. As previously mentioned, the lower-order fractional derivative represents the epidemic peak. Finally, against the backdrop of the vaccination game utilizing fractional-order derivative models, individual consciousness plays a crucial role in effectively negotiating quarantine and isolation regulations. When making strategic decisions on vaccination, people should also consider their degree of understanding of public health policies. Quarantine and isolation are crucial tactics for managing the transmission of contagious diseases, especially when dealing with newly emerged variations. Governments/policymakers can precisely adjust quarantine and isolation rules to compromise public health goals and individual liberties using fractional-order derivative modeling tools. Increased individual consciousness promotes adherence to these measures, thereby improving the efficacy of vaccination efforts and eventually aiding the collective endeavor to reduce the burden of infectious diseases.

This part represents the proposed models’ overall disease dynamics through a surface diagram (Figs. 14, 15, 16, 17, 18, 19 and 20). It is well established that a surface diagram is a very effective visualization technique used to illustrate the progression of a disease over time, taking into account its fractional order $\alpha$, which ranges from 0 to 1. One can detect complex disease dynamics by graphing the disease’s evolution (infection), vaccination coverage (VC), policy (quarantine and isolation), and final epidemic size (FES) ($z$-axis) on a three-dimensional surface, with time on one axis ($y$-axis) and fractional order on another ($x$-axis). As the fractional order ranges from 0 to 1, representing the level of complexity in disease dynamics, the surface contours depict the progression of the disease, VC, policy, and FES over time, capturing subtle details of its spread, severity, and responsiveness to interventions. This representation allows researchers and healthcare professionals to learn about the complex relationship between time, the fractional order of the disease, and its impact on populations. It helps to enhance comprehension of disease dynamics and assists in creating precise intervention strategies. Here, Figs. 14, 15, 16, 17, 18, 19 and 20 demonstrate the impact of government forces in participating in vaccination programs, personal awareness, quarantine policy, quarantine policy with personal awareness, isolation policy, isolation policy with personal awareness, and combined policy of quarantine and isolation, government force and personal awareness to elucidate the overall dynamics of infected, vaccinated, quarantine or isolated and recovered individuals. Figure 20, subfigure A, represents the lower order fractional derivative that shows the epidemic peak.

The waning of artificial and natural immunity presents substantial challenges for people in many situations. Waning immunity in the infected individuals increases the likelihood of reinfection, which can result in more severe sickness or consequences. Vaccinated people can undergo waning immunity over time, requiring booster doses to maintain adequate levels of protection. Individuals who have been isolated are in danger of being exposed to the virus again when they reintegrate into society, significantly if their immunity has decreased. Meanwhile, persons who have been cured may decline their immunity, making them susceptible to becoming infected again. Within the framework of vaccination campaigns, the expense and effectiveness of vaccines emerge as pivotal elements in ascertaining the accessibility and degree of safeguarding provided to the population. The increased costs of vaccination restrict access, worsening existing inequalities, while the varied effectiveness of vaccines can impact public confidence and willingness to comply with immunization initiatives. Hence, resolving declining immunity necessitates a comprehensive method that includes vaccination strategies, public health measures, and continuous research to guarantee enduring safeguards against contagious diseases. Thus, for the first time, we represent the fractional-order phase-portrayed trajectory graph Figs. 21, 22, 23, 24, 25, 26, 27, 28, 29, 30, 31 and 32 with different settings of vaccination costs and efficacy ${(C}_{V},\eta )=\left(\mathrm{0.9,0.1}\right),(\mathrm{0.5,0.5}) {\text{and}} (\mathrm{0.1,0.9})$ to demonstrate the immunity-waiting scenarios of infected, vaccinated, quarantined, isolated, and recovered individuals. Naturally, when artificial immunity wanes at a certain rate, people are interested in natural recovery (Figs. 24, 28, 32), protecting themselves from pathogens through personal awareness and non-pharmaceutical interventions, namely, quarantine and isolation policy (Figs. 23, 27, 31). Opposite scenarios when natural immunity wanes at a certain rate. Again, vaccination cost and efficacy play a significant role in this situation. Therefore, when both immunities wane at a specific rate, people generally are in dilemma situations. As a result, a susceptible free rider situation arises, and more precisely, a certain portion of the total population did not show interest in taking any protection measures, which is one of the most hazardous scenarios of an epidemic.

## Conclusion

In conclusion, our novel ABC fractional-order epidemic modeling combined with evolutionary game theory (EGT) offers significant insights into the efficacy of personal protection, quarantine, and isolation measures in combating epidemic diseases. The objective was to examine the interplay of these three regulations in controlling the transmission of diseases with vaccination games and government forces to participate in vaccination programs. We have shown the complex interactions between disease transmission and containment techniques via a comparative study, emphasizing the significance of flexible and diverse approaches in public health policy. We observed that the disease curve demonstrates a decreasing trend with a slowing of the epidemic peak due to the increasing personal awareness parameter values rate. A higher quarantine rate decreases the disease curve and final epidemic size (FES) and increases vaccination coverage (VC), and a higher rate of perfect isolation policy alleviates the disease outbreak and allows individuals to participate in vaccination campaigns. The combined strategy with the higher value of vaccine efficacy demonstrates one of the fundamental characteristics of fractional-order derivatives, where deterministic processes fail to describe such scenarios that the lower order fractional-order derivative shows the highest peak of the epidemic, and the disease almost dies out. The government forces would not work significantly if the vaccine efficacy is not high. According to the cost-effectiveness analysis, we observed that the lower price of vaccines motivated individuals to participate in vaccination programs. Finally, we illustrated the artificial and natural immunity waning scenarios to represent the dilemma of humans through three vaccination cost and efficacy settings. Therefore, the current study combines mathematical modeling with game theory to enhance comprehension of epidemic dynamics and provide practical guidance for policymakers and public health authorities in creating efficient intervention methods to protect public health and well-being.

## Data Availability

All data generated or analyzed during this study are included in this article.
